# Prevalence, characteristics, and projection of long-term childhood cancer survivors in Sweden

**DOI:** 10.1007/s10654-026-01394-2

**Published:** 2026-05-07

**Authors:** Elena Extrand, Emerald G. Heiland, Genevieve Allen, Mia Giertz, Hanna Mogensen, Hannah L. Brooke

**Affiliations:** 1 Medical Epidemiology, Department of Surgical Sciences, Uppsala University, Uppsala, Sweden; 2 Department of Women and Children’s Health, Uppsala University, Uppsala, Sweden; 3 Department of Immunology, Genetics and Pathology, Cancer Precision Medicine, Uppsala University, Uppsala, Sweden

**Keywords:** Prevalence, Pediatrics, Neoplasms, Child, Epidemiology, Cancer survivors

## Abstract

Population-level descriptions of long-term childhood cancer survivors are fundamental to survivorship care and research but seldom available. Accordingly, we aimed to describe long-term childhood cancer survivors at the population-level and project future prevalence. In this register-based study we calculated the absolute number and prevalence proportions of all individuals diagnosed with a childhood cancer (aged 0–14 years, 1958–2018) in Sweden who survived ≥ 5 years post-diagnosis and were alive and residing in Sweden on December 31st, 2023. We also described the clinical and sociodemographic characteristics of this population and presented the observed prevalence over time (1990–2023) and projected prevalence under different mortality assumptions (2024–2040). On December 31st, 2023, there were 8645 long-term childhood cancer survivors in Sweden, equivalent to nearly 1 in 1000 inhabitants (921 persons per million). Leukemias (28.3%) and central nervous system tumors (27.0%) were the most common childhood cancer diagnoses, although the distribution of cancer type varied by attained age. Disease burden in the preceding five years was heterogeneous: approximately 25–30% of the survivors had no recent diagnoses or prescriptions, while a similar proportion experienced substantial morbidity. Most adult survivors were employed (72.0%) and relatively few received sickness benefits (9.7%). From 1990 to 2023, the long-term survivor population tripled in size. Projected mean annual growth was between 1.6% and 2.2%, with the population increasing to approximately 11,400 − 12,600 individuals by 2040. As this heterogeneous population continues growing, our comprehensive description can help plan survivorship care and provide a benchmark for prevalence estimates in settings with less complete data.

**Supplementary Information:**

The online version contains supplementary material available at 10.1007/s10654-026-01394-2.

## Introduction

Globally, approximately 400,000 children (ages 0–14 years) are diagnosed with cancer each year [1]. In Europe, a large majority now survive their cancer treatment [2]. However, as a result of their disease and treatments, survivors face an increased risk of hospitalization [3] and mortality [4], a high burden of chronic health conditions [5, 6], and difficulties with employment, education, and family formation [7]. Accordingly, long-term follow-up care is recommended to prevent, detect, and treat the adverse effects of cancer treatment [8]. To plan for this care and inform future research, understanding the size, composition, and trajectory of the population of long-term (at least five years post-diagnosis) childhood cancer survivors (CCS) is essential.

A growing body of cohort studies has provided invaluable insights into the long-term sequelae of childhood cancer [3–7, 9]. However, because these studies typically focus on relative risks and often make exclusions aimed at reducing bias in effect estimates, they are less useful for understanding the composition and characteristics of the long-term CCS population as a whole. To convey the magnitude of outcomes and capture heterogeneity, descriptive studies emphasizing absolute numbers at the population level are indispensable. Yet, such studies remain scarce.

Robust descriptions of prevalence are especially fundamental to survivorship research and care. However, estimating the complete prevalence of CCS demands historical data that few sources can provide, in Europe or elsewhere [10]. Existing studies of CCS prevalence use cancer registers with limited population coverage or surveillance periods that are too short to capture individuals diagnosed many decades ago [10–13]. Consequently, prevalence estimates frequently reflect only a subset of the population and are dependent on modeling assumptions to account for earlier diagnoses or missing data.

Understanding temporal trends in CCS prevalence is also critical for planning long-term survivorship care. Since childhood cancer survival rates have improved dramatically in recent decades across many contexts [2, 14, 15], it is reasonable to expect that the long-term CCS population has grown in tandem. Studies examining temporal prevalence trends support this notion, though the magnitude of increase varies by context and methodology [12, 13, 16, 17]. Looking to the future, projections of CCS prevalence remain limited to North America [15, 18]. Little is known about how the size of the CCS population will evolve elsewhere.

In Sweden, where nationwide data have been available on cancer diagnoses since 1958 [19], many of the limitations affecting previous prevalence estimates can be overcome. In this study, we leverage Sweden’s long-standing tradition of cancer registration to provide a comprehensive description of long-term CCS at the population-level. This includes a description of the prevalence of long-term CCS and their clinical and sociodemographic characteristics on December 31st, 2023, a temporal description of prevalence from 1990 to 2023, and projections of prevalence from 2024 to 2040 under different mortality scenarios.

## Materials and methods

### Participants

All individuals diagnosed with a malignant childhood cancer (diagnosed ages 0–14 years) in Sweden between 1958 and 2018 were identified using the National Cancer Register (Online Resource Fig. 1). To be included, individuals must have been registered as living in Sweden on December 31st in at least one year between 1990 and 2019. The National Cancer Register, which includes mandatory nationwide registration of all malignancies and some benign tumors since 1958, is considered to be practically complete [19, 20]. Morphology and topography codes were used to classify childhood cancer into 12 groups according to the International Classification of Childhood Cancer (ICCC-3-2017) [21, 22]. Cases diagnosed before 2005 were classified to mimic these groups. Certain cancers, such as central nervous system (CNS) tumors, were considered malignant by site (Online Resource Methods 1).

To define the population of long-term CCS alive and residing in Sweden on December 31st of each year from 1990 to 2023, individuals who were less than five years post-diagnosis, had emigrated from Sweden (without subsequent re-immigration), or who had died on or before December 31st of that year were excluded. Short-term (less than five years post-diagnosis) CCS were excluded to ensure that the study population represented individuals likely to require long-term survivorship care. This approach also minimized the possibility that the sociodemographic and clinical characteristics of CCS still in the acute treatment phase would obscure the characteristics of long-term CCS.

### Data sources and variables

We linked individuals across several national population-based registers using Swedish personal identity numbers [23]. Additional, aggregate data on the size and composition of the total Swedish population aged 5–79 was extracted to identify the population at risk (necessary for calculating prevalence proportions) [24].

#### Clinical characteristics

The National Cancer Register provided data on individuals’ first malignant cancer diagnosis, including age at diagnosis (< 1, 1–4, 5–9, 10–14 years), year of diagnosis (categorized into decades), time since diagnosis (5–9, 10–19, 20–29, 30–39, ≥ 40 years), and cancer type. Due to small cell counts, ICCC-3-2017 groups IV–XII were combined in some analyses.

The Total Population Register provided data on sex and attained age on December 31st, 2023, (categorized as 5–18, 19–25, 26–35, 36–45, 46–55, > 55 years). National Patient Register data were used to determine whether an individual had at least one inpatient or outpatient specialist care visit in 2023 (yes/no) [25, 26]. The National Prescribed Drug Register provided the number (0, 1–2, 3–4, 5–9, ≥ 10) of unique drug classes dispensed in 2022, defined using ATC codes at the chemical subgroup (4th) level [27].

The recent disease burden among long-term CCS was captured using primary and secondary diagnoses (positions 1–30) in inpatient and outpatient specialist care recorded in the National Patient Register in the five years preceding December 31st, 2023. The register contains complete nationwide information on both inpatient and outpatient specialist care; it does not include primary care visits [25, 26]. A dichotomous variable was constructed for each organ-specific disease grouping/sub-grouping using Swedish ICD-10 codes (Online Resource Methods 2), based on previously published disease groupings relevant to CCS [3, 28]. An additional categorical variable summarized the number of involved organ systems (0, 1–2, ≥ 3).

#### Sociodemographic characteristics

Sociodemographic characteristics were captured in 2023 (unless otherwise stated) using the Total Population Register and the Longitudinal Integrated Database for Health Insurance and Labour Market Studies [24, 29]. Urbanicity was defined as residing predominately outside (rural) or within/adjacent to (urban) population concentrations [30]. Migration background was categorized as Swedish (two Swedish-born parents), second-generation (born in Sweden with at least one foreign-born parent), or first-generation (born abroad with at least one foreign-born parent). Region of residence was categorized according to Sweden’s six cancer care regions [31]. For individuals aged < 15 years in 2023, the mother’s region served as a proxy.

The following sociodemographic characteristics were only assessed among adults (aged > 18 years on December 31st, 2023). Civil status was classified as unmarried, divorced/widowed, or married/in a registered partnership. Number of children (0, 1, 2, ≥ 3) included both biological and adoptive children. Labor market status in 2022 was categorized as employed, unemployed, on sickness/disability leave, or other. Education reflects the highest level of educational attainment (lower secondary or less, upper secondary, university studies, or post-graduate studies). Income was defined as annual household disposable income in 2022 and divided into quartiles; quartile thresholds were derived from the entire Swedish population, accounting for attained age and household size.

#### Temporal and projected prevalence

The observed (1990–2023) annual prevalence of long-term CCS was defined as the number of long-term CCS alive and residing in Sweden on December 31st of each year. The absolute number of long-term CCS from 2024 to 2040 was projected by simulating the inflow and outflow of individuals from the observed population in 2023. Inflow was computed using information on the underlying population at risk, childhood cancer incidence rates, and short-term (0–5 years post-diagnosis) survival rates among children diagnosed with cancer. Incidence rates were held stable across the projection period, based on recent national trends [32]. Two alternate short-term survival scenarios were generated. One assumed recent survival rates plateaued across the projection period; the second assumed survival rates improved continuously at historical rates. Outflow was quantified using data on migration and long-term mortality. Two alternate scenarios of long-term mortality were explored. One assumed long-term CCS experienced similar mortality rates as the general population; the other was based on historical trends in excess mortality among long-term CCS [4]. Detailed information on data sources and definitions underlying the projections are in the Online Resources (Online Resource Methods 3).

#### Statistical analyses

Prevalence counts were calculated as the sum of all individuals who met eligibility criteria. Proportions were calculated by dividing the absolute count of long-term CCS by the population at risk in five-year age intervals. Individuals aged 65–79 years were grouped together because this age band only has partial coverage in the register. Prevalence counts and proportions were also reported by age at diagnosis, sex, and attained age. Frequency tables were generated to illustrate clinical and sociodemographic characteristics, both overall and stratified by cancer type, year of diagnosis (1958–1990 vs. 1991–2018), and attained age in 2023.

Prevalence from 2024 to 2040 was estimated using an iterative, stepwise projection model based on annual inflow and outflow of long-term CCS in Sweden. Four discrete projections were modeled by combining the two alternate short-term survival and long-term mortality scenarios. Detailed methodology is provided in the Online Resources (Online Resource Methods 4).

Analyses were conducted in Stata v18.5 (StataCorp., College Station, TX, USA) and R v4.3.1.

## Results

### Prevalence

As of December 31st, 2023, there were 8645 long-term CCS (diagnosed in Sweden aged 0–14 years, 1958–2018, survived at least five years post-diagnosis) living in Sweden (Online Resource Fig. 1). Accordingly, the prevalence proportion of long-term CCS was 921 persons per million, or 1 long-term CCS per 1086 individuals in the general population. Across most age bands, prevalence proportions were higher among males than females. Prevalence was highest among males ages 20–24 years (1666 long-term CCS per million persons). Prevalence counts and proportions stratified by sex, age at diagnosis, and attained age are shown in the Online Resources (Online Resource Tables 1 and 2).

### Clinical characteristics

Most long-term CCS had a history of leukemias (28.3%), CNS tumors (27.0%), or lymphomas (10.7%) (Table 1). Nearly half (46.7%) were diagnosed before age five and approximately two-thirds (67.5%) were diagnosed from 1990 onward. Median time since diagnosis was 25 years (Interquartile range: 14–38 years) (Online Resource Table 3). In the last year, most had attended one or more outpatient specialist visits (64.4%) or obtained one or more prescriptions (68.8%) (Table 2). Fewer had undergone inpatient hospital care (10.0%) or filled prescriptions for five or more unique drug classes (22.9%). When examining healthcare utilization across age subsets within the long-term CCS population, having attended one or more outpatient specialist visits was most common among long-term CCS aged 5–18 years (89.4%). By comparison, having undergone inpatient hospital care (14.8%) or having filled prescriptions for five or more unique drug classes (43.0%) was most common among the oldest long-term CCS (aged > 55 years).

**Table 1:** Clinical characteristics of long-term childhood cancer survivors (diagnosed ages 0–14, 1958–2018, survived ≥ 5 years from diagnosis) registered as alive and living in Sweden on December 31st, 2023 (*N* = 8,645)

	*N*	%
**Age on December 31st, 2023 (years)**^a^		
5–18	1,705	19.7
19–25	1,349	15.6
26–35	2,002	23.2
36–45	1,518	17.6
46–55	1,187	13.7
>55	884	10.2
**Age at diagnosis (years)**^a^		
<1	900	10.4
1–4	3,137	36.3
5–9	2,229	25.8
10–14	2,379	27.5
**Year of diagnosis**^a^		
1958–1969	537	6.2
1970–1979	852	9.9
1980–1989	1,428	16.5
1990–1999	1,884	21.8
2000–2009	1,890	21.9
2010–2018	2,054	23.8
**Time since diagnosis (years)**^a^		
5–9	1,139	13.2
10–19	2,050	23.7
20–29	1,920	22.2
30–39	1,657	19.2
≥40	1,879	21.7
**Cancer type**^a, b^		
Leukemias		
*ALL*	2,035	23.5
*AML*	263	3.0
*Other leukemias*	159	1.8
Lymphomas		
*Hodgkin lymphomas*	312	3.6
*Non-Hodgkin lymphomas*	176	2.0
*Other lymphomas*	444	5.1
CNS tumors		
*Ependymomas and choroid plexus tumors*	198	2.3
*Astrocytomas and other gliomas*	1,101	12.7
*Embryonal tumors*	346	4.0
*Other CNS tumors*	691	8.0
Neuroblastomas and PNS	281	3.3
Retinoblastomas	326	3.8
Renal tumors	596	6.9
Hepatic tumors	92	1.1
Malignant bone tumors	249	2.9
Soft tissue sarcomas	435	5.0
Germ cell tumors	281	3.3
Epithelial tumors	444	5.1
Other and unspecified	41	0.5
Unclassifiable	175	2.0

*ALL* Acute lymphoid leukemia, *AML* Acute myeloid leukemia, *CNS* Central nervous system, *PNS* Peripheral nervous system

^a^Describe an individual’s first cancer diagnosis

^b^ Cancer type is classified according to the International Classification of Childhood Cancer, third edition, 2017 update. Groups I-III are broken down into sub-groups, the remainder (groups IV-XII) are presented at the group level

**Table 2:** Healthcare utilization in the last year among long-term childhood cancer survivors (diagnosed ages 0–14, 1958–2018, survived ≥ 5 years from diagnosis) registered as alive and living in Sweden on December 31st, 2023 (*N* = 8,645)

	Age on December 31st, 2023 (years)									
	5–18		19–35		36–55		>55		Overall	
	N	%	N	%	N	%	N	%	N	%
**Inpatient visit**										
No	1,539	90.3	3,019	90.1	2,466	91.2	753	85.2	7,777	90.0
Yes	166	9.7	332	9.9	239	8.8	131	14.8	868	10.0
**Outpatient visit**										
No	181	10.6	1,337	39.9	1,208	44.7	355	40.2	3,081	35.6
Yes	1,524	89.4	2,014	60.1	1,497	55.3	529	59.8	5,564	64.4
**Number of unique prescribed drug classes**										
0	678	39.8	1,132	33.8	739	27.3	152	17.2	2,701	31.2
1–2	527	30.9	1,055	31.5	778	28.8	187	21.2	2,547	29.5
3–4	255	15.0	549	16.4	449	16.6	165	18.7	1,418	16.4
5–9	191	11.2	459	13.7	516	19.1	250	28.3	1,416	16.4
≥10	54	3.2	156	4.7	223	8.2	130	14.7	563	6.5

Unique prescribed drugs classes is measured in 2022, while outpatient and inpatient visits are measured in 2023

**Fig. 1:**
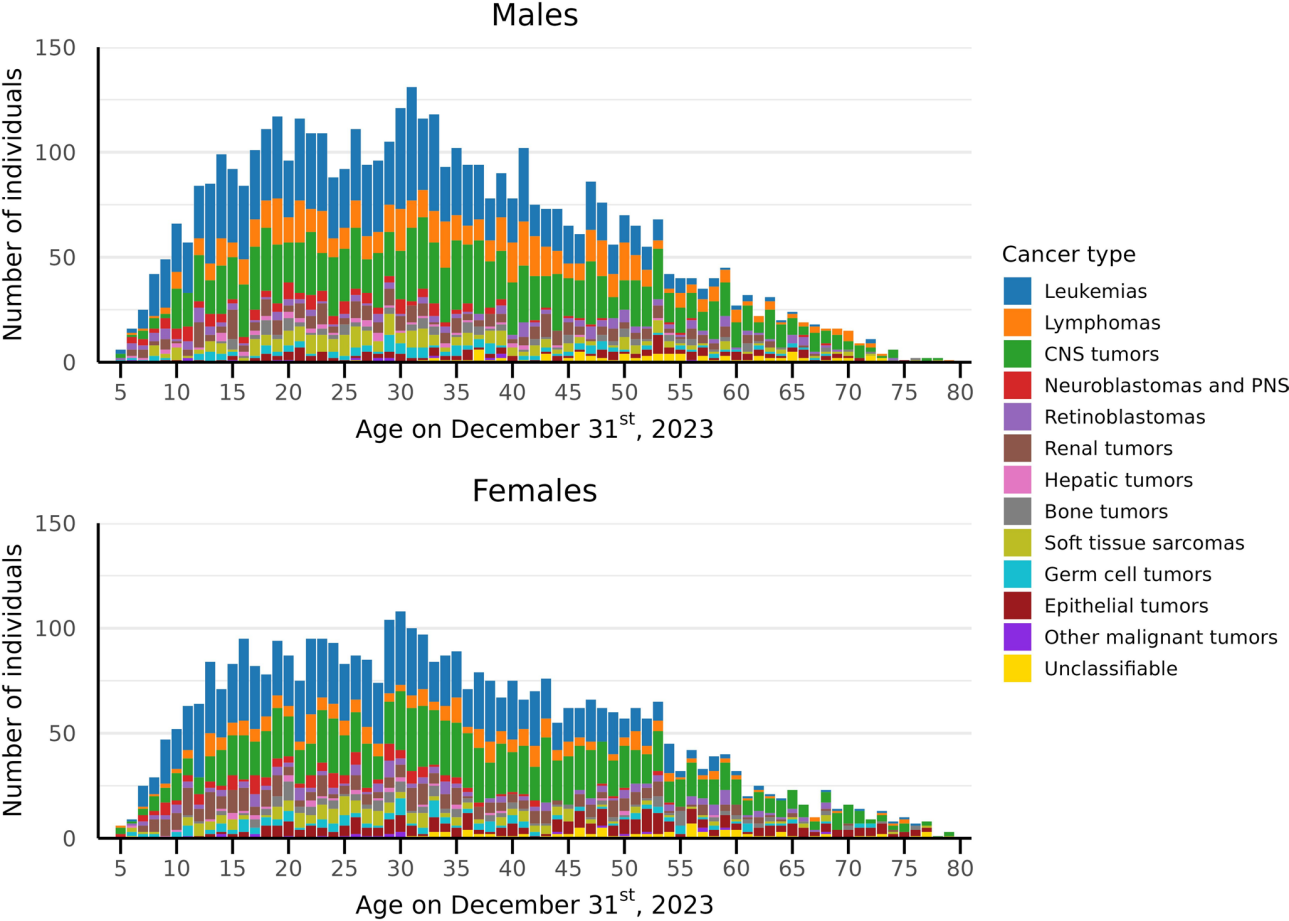
Distribution of cancer type and age among the population of long-term childhood cancer survivors (diagnosed at ages 0–14 years, from 1958–2018, ≥ 5 years post-diagnosis) registered as living in Sweden on December 31st, 2023, by sex. Cancer type is classified according to the International Classification of Childhood Cancer, third edition, 2017 update. CNS = Central Nervous System, PNS = Peripheral Nervous System. All figures were generated using R version 4.3.1

As illustrated in Fig. 1, the distribution of cancer types differed across age subsets within the long-term CCS population. Leukemias were more common among younger long-term CCS, representing approximately one-third (33.8%) of those aged 35 years or younger, but merely 6.2% of the oldest long-term CCS (aged > 55 years). The most prevalent cancer types among the oldest long-term CCS (aged > 55 years) were CNS tumors (36.1%) and epithelial tumors (13.9%).

The recent disease burden among long-term CCS was heterogeneous. Approximately one-quarter (25.1%) had no diagnosed diseases within outpatient specialist or inpatient care in the past five years, 42.3% had one or two, and 32.6% had three or more (Table 3). Diseases of the eyes or ears were the most prevalent (24.0%), largely due to inflammatory and other eye conditions. Endocrine diseases, nutritional deficiencies and other metabolic diseases (22.7%), diseases of the urinary system and genital organs (21.3%), and diseases of the bones, joints, and muscles (19.8%) were also common. Prevalence of disease sub-groupings are available in the Online Resources (Online Resource Tables 4, 5, 6, 7, 8, 9, 10, 11, 12, 13, 14 and 15).

**Table 3:** Disease burden based on primary and secondary diagnoses recorded in hospital inpatient and outpatient specialist care in the five-year period preceding December 31st, 2023 among long-term childhood cancer survivors (diagnosed ages 0–14, 1958–2018, survived ≥ 5 years from diagnosis) registered as alive and living in Sweden on December 31st, 2023 (*N* = 8,645)

	*N*			%
**Number of diseases**^a^				
0	2,172			25.1
1–2	3,655			42.3
≥ 3	2,818			32.6
**Diagnosis**^b^				
Infectious or parasitic diseases	922			10.7
Benign tumors	935			10.8
Blood diseases	408			4.7
Endocrine, nutritional, or metabolic diseases	1,965			22.7
Mental or behavioral diseases	1,701			19.7
Diseases of the nervous system	1,395			16.1
Diseases of the eye or ear	2,078			24.0
Diseases of the circulatory system	1,048			12.1
Diseases of the respiratory system	1,149			13.3
Diseases of the digestive system	1,286			14.9
Diseases of the urinary system or genitals	1,838			21.3
Diseases of the skin and subcutaneous tissues	1,380			16.0
Diseases of the bones, joints, and muscles	1,715			19.8

^a^Column percentage

^b^Row percentage, i.e. proportion of the total long-term childhood cancer survivor population with a diagnosis within each category

**Fig. 2:**
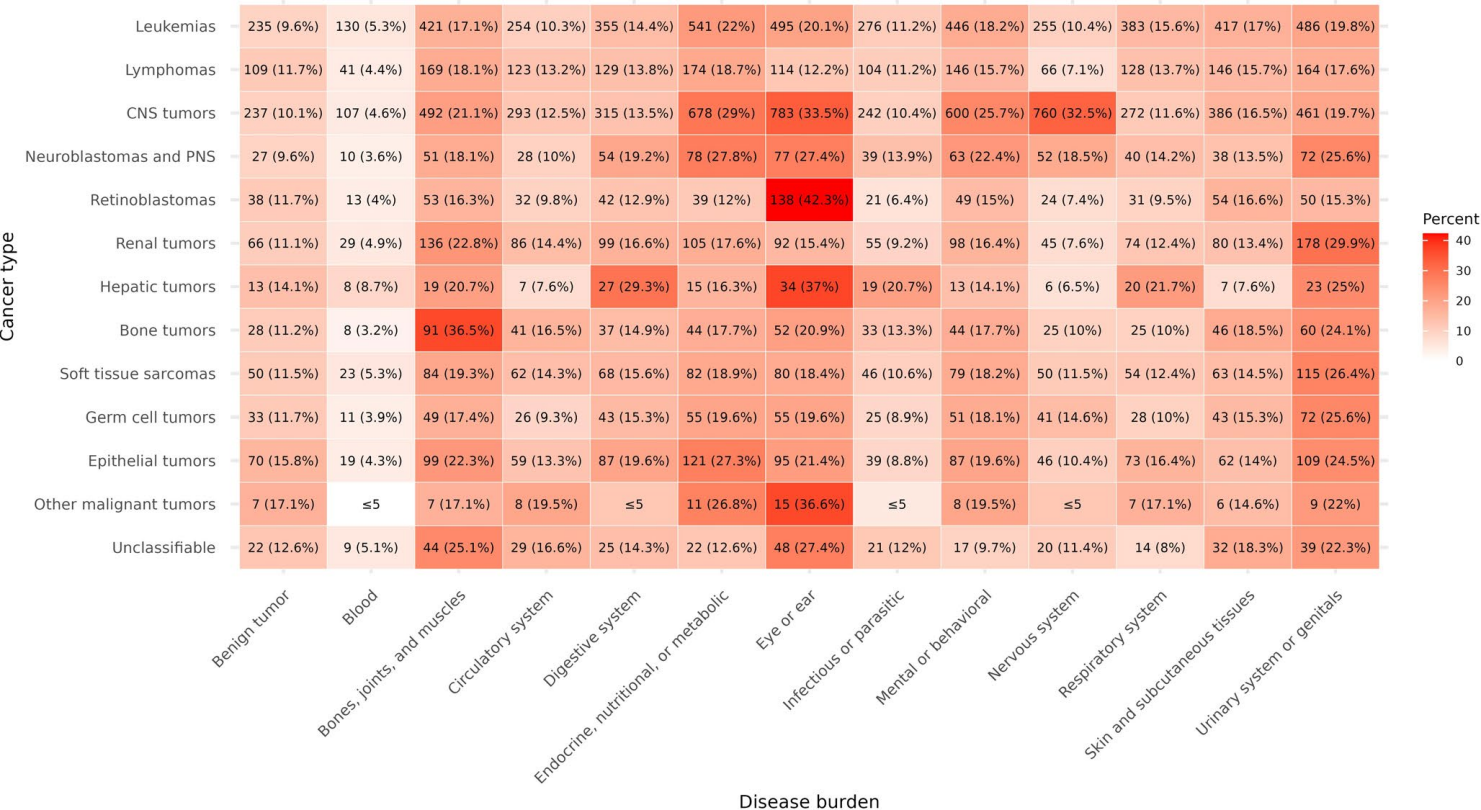
Recent disease burden by cancer type among the population of long-term childhood cancer survivors (diagnosed at ages 0–14 years, from 1958–2018, ≥ 5 years post-diagnosis) registered as living in Sweden on December 31st, 2023. Cells display absolute counts and proportions. A darker shade reflects a higher prevalence of diagnosed disease. Cancer type is classified according to the 3rd edition of the International Classification of Childhood Cancer, 2017 update. Disease burden reflects diagnoses recorded in outpatient specialist care and inpatient care in the five-year period preceding December 31st, 2023. CNS = Central Nervous System, PNS = Peripheral Nervous System. All figures were generated using R version 4.3.1

Disease burden varied by cancer type (Fig. 2, Online Resource Tables 16 and 17) and by year of diagnosis (Online Resource Tables 16 and 17). Diseases of the eyes or ears were the most prevalent among long-term CCS with a history of retinoblastomas (42.3%), hepatic tumors (37.0%), other malignant tumors (36.6%), and CNS tumors (33.5%). With the exception of mental and behavioral diseases and circulatory diseases, the proportion of long-term CCS with diagnoses in each disease group were generally similar by year of diagnosis (1958–1990 vs. 1991–2018). Mental or behavioral diagnoses were recorded in 13.2% of long-term CCS diagnosed from 1958 to 1990, compared to 23.1% of those diagnosed from 1991 to 2018. Conversely, diseases of the circulatory system were reported in 21.0% of long-term CCS diagnosed from 1958 to 1990 and 7.4% of those diagnosed from 1991 to 2018.

### Sociodemographic characteristics

As demonstrated in Table 4, slightly more long-term CCS were male (53.3%) than female. Most were of Swedish background (77.7%) and lived in urban areas (85.6%). Among adult (age > 18 years) long-term CCS, most were employed (72.0%) and had an upper secondary (44.7%) or university (31.9%) education. Generally, adult long-term CCS were uniformly distributed across the general population income quartiles. One-quarter of adult long-term CCS (24.2%) were married and 42.6% had one or more children. The sociodemographic characteristics of long-term CCS diagnosed from 1958 to 1990 differed from those diagnosed between 1991 and 2018. Most adult long-term CCS diagnosed from 1958 to 1990 had children (64.8%) and many were married (39.3%). Among adult long-term CCS diagnosed from 1991 to 2018, a smaller proportion had children (25.8%) and were married (12.9%). A migrant background (first- or second-generation) was more common among long-term CCS diagnosed in 1991–2018 (25.6%) than in 1958–1990 (15.9%).

**Table 4:** Sociodemographic characteristics of long-term childhood cancer survivors (diagnosed ages 0–14, 1958–2018, survived ≥ 5 years from diagnosis) registered as alive and living in Sweden on December 31st, 2023, by cancer type and year of diagnosis (1958–1990 vs. 1991–2018) (*N* = 8,645)

	Diagnosed 1958–1990					Diagnosed 1991–2018					
	Leukemias	Lymphomas	CNS	Other	All cancers	Leukemias	Lymphomas	CNS	Other	All cancers	Overall
	N (%)	N (%)	N (%)	N (%)	N (%)	N (%)	N (%)	N (%)	N (%)	N (%)	N (%)
**Sex**											
Male	292 (48.0)	219 (69.5)	452 (49.8)	552 (48.0)	1515 (50.8)	1037 (56.1)	413 (66.9)	794 (55.6)	851 (48.1)	3095 (54.6)	4610 (53.3)
Female	316 (52.0)	96 (30.5)	456 (50.2)	597 (52.0)	1465 (49.2)	812 (43.9)	204 (33.1)	634 (44.4)	920 (51.9)	2570 (45.4)	4035 (46.7)
**Migration background**											
1st generation	12 (2.0)	13 (4.1)	22 (2.4)	26 (2.3)	73 (2.4)	66 (3.6)	40 (6.5)	58 (4.1)	59 (3.3)	223 (3.9)	296 (3.4)
2nd generation	78 (12.8)	51 (16.2)	108 (11.9)	164 (14.3)	401 (13.5)	448 (24.2)	134 (21.7)	268 (18.8)	379 (21.4)	1229 (21.7)	1630 (18.9)
Swedish background	518 (85.2)	251 (79.7)	778 (85.7)	959 (83.5)	2506 (84.1)	1335 (72.2)	443 (71.8)	1102 (77.2)	1333 (75.3)	4213 (74.4)	6719 (77.7)
**Cancer care region**^a, b^											
Capital	115 (18.9)	76 (24.1)	182 (20.0)	282 (24.5)	655 (22.0)	438 (23.7)	142 (23.0)	312 (21.8)	467 (26.4)	1359 (24.0)	2014 (23.3)
Central	132 (21.7)	58 (18.4)	195 (21.5)	222 (19.3)	607 (20.4)	353 (19.1)	133 (21.6)	255 (17.9)	310 (17.5)	1051 (18.6)	1658 (19.2)
Southeast	61 (10.0)	31 (9.8)	94 (10.4)	125 (10.9)	311 (10.4)	208 (11.2)	58 (9.4)	181 (12.7)	181 (10.2)	628 (11.1)	939 (10.9)
South	120 (19.7)	55 (17.5)	168 (18.5)	208 (18.1)	551 (18.5)	314 (17.0)	109 (17.7)	269 (18.8)	318 (18.0)	1010 (17.8)	1561 (18.1)
West	119 (19.6)	71 (22.5)	160 (17.6)	216 (18.8)	566 (19.0)	369 (20.0)	130 (21.1)	288 (20.2)	367 (20.7)	1154 (20.4)	1720 (19.9)
North	61 (10.0)	24 (7.6)	109 (12.0)	96 (8.4)	290 (9.7)	167 (9.0)	45 (7.3)	123 (8.6)	128 (7.2)	463 (8.2)	753 (8.7)
**Urbanicity**^b^											
Rural	106 (17.4)	45 (14.3)	119 (13.1)	200 (17.4)	470 (15.8)	257 (13.9)	92 (14.9)	205 (14.4)	224 (12.6)	778 (13.7)	1248 (14.4)
Urban	502 (82.6)	270 (85.7)	789 (86.9)	949 (82.6)	2510 (84.2)	1,592 (86.1)	525 (85.1)	1223 (85.6)	1547 (87.4)	4887 (86.3)	7397 (85.6)
**Household income quartile**^c, d^											
Q1 (lowest)	159 (26.2)	67 (21.3)	360 (39.6)	249 (21.7)	835 (28.0)	292 (24.0)	106 (22.3)	299 (28.3)	280 (23.1)	977 (24.7)	1812 (26.1)
Q2	180 (29.6)	73 (23.2)	219 (24.1)	265 (23.1)	737 (24.7)	291 (23.9)	111 (23.4)	258 (24.4)	279 (23.0)	939 (23.7)	1676 (24.1)
Q3	136 (22.4)	88 (27.9)	187 (20.6)	305 (26.5)	716 (24.0)	325 (26.7)	131 (27.6)	250 (23.7)	292 (24.1)	998 (25.2)	1714 (24.7)
Q4 (highest)	133 (21.9)	87 (27.6)	142 (15.6)	330 (28.7)	692 (23.2)	309 (25.4)	127 (26.7)	249 (23.6)	361 (29.8)	1,046 (26.4)	1738 (25.0)
**Education**^c^											
Lower secondary	59 (9.7)	27 (8.6)	142 (15.6)	106 (9.2)	334 (11.2)	172 (14.1)	56 (11.8)	201 (19.0)	144 (11.9)	573 (14.5)	907 (13.1)
Upper secondary	275 (45.2)	140 (44.4)	435 (47.9)	487 (42.4)	1337 (44.9)	524 (43.1)	224 (47.2)	466 (44.1)	553 (45.6)	1767 (44.6)	3104 (44.7)
University studies	195 (32.1)	100 (31.7)	242 (26.7)	384 (33.4)	921 (30.9)	406 (33.4)	154 (32.4)	321 (30.4)	411 (33.9)	1292 (32.6)	2213 (31.9)
Post-graduate	79 (13.0)	48 (15.2)	89 (9.8)	172 (15.0)	388 (13.0)	115 (9.4)	41 (8.6)	68 (6.4)	104 (8.6)	328 (8.3)	716 (10.3)
**Labor market status**^c, d^											
Employed	502 (82.6)	255 (81.0)	584 (64.3)	901 (78.4)	2242 (75.2)	850 (69.8)	362 (76.2)	666 (63.1)	875 (72.2)	2753 (69.5)	4995 (72.0)
Unemployed	22 (3.6)	6 (1.9)	28 (3.1)	33 (2.9)	89 (3.0)	46 (3.8)	16 (3.4)	60 (5.7)	38 (3.1)	160 (4.0)	249 (3.6)
Sickness leave	52 (8.6)	23 (7.3)	184 (20.3)	75 (6.5)	334 (11.2)	88 (7.2)	13 (2.7)	166 (15.7)	72 (5.9)	339 (8.6)	673 (9.7)
Other	32 (5.3)	31 (9.8)	112 (12.3)	140 (12.2)	315 (10.6)	233 (19.1)	84 (17.7)	164 (15.5)	227 (18.7)	708 (17.9)	1023 (14.7)
**Civil status**^c^											
Married	229 (37.7)	143 (45.4)	286 (31.5)	513 (44.6)	1171 (39.3)	140 (11.5)	74 (15.6)	149 (14.1)	147 (12.1)	510 (12.9)	1681 (24.2)
Divorced/Widowed	61 (10.0)	44 (14.0)	121 (13.3)	169 (14.7)	395 (13.3)	22 (1.8)	18 (3.8)	21 (2.0)	21 (1.7)	82 (2.1)	477 (6.9)
Unmarried	318 (52.3)	128 (40.6)	501 (55.2)	467 (40.6)	1414 (47.4)	1055 (86.7)	383 (80.6)	886 (83.9)	1044 (86.1)	3368 (85.1)	4782 (68.9)
**Number of children**^c^											
No children	216 (35.5)	98 (31.1)	417 (45.9)	319 (27.8)	1050 (35.2)	908 (74.6)	316 (66.5)	801 (75.9)	912 (75.2)	2937 (74.2)	3987 (57.4)
1	102 (16.8)	40 (12.7)	125 (13.8)	187 (16.3)	454 (15.2)	125 (10.3)	63 (13.3)	104 (9.8)	110 (9.1)	402 (10.2)	856 (12.3)
2	207 (34.0)	116 (36.8)	248 (27.3)	416 (36.2)	987 (33.1)	150 (12.3)	73 (15.4)	123 (11.6)	147 (12.1)	493 (12.4)	1480 (21.3)
3 or more	83 (13.7)	61 (19.4)	118 (13.0)	227 (19.8)	489 (16.4)	34 (2.8)	23 (4.8)	28 (2.7)	43 (3.5)	128 (3.2)	617 (8.9)

To protect confidentiality and minimize disclosure risk, some categories have been combined. Individuals with missing data were included in the largest relevant category as part of this approach. Cancer type is classified according to the International Classification of Childhood Cancer, third edition, 2017 update; groups IV-XII are combined into *Other.* CNS = Central Nervous System tumors

^a^Capital region includes Gotland

^b^For survivors ages 5–18 in 2023, the mother’s region was used as a proxy

^c^Only tabulated for participants aged 19 years or older as of December 31st, 2023

^d^Measured in 2022

Among adult long-term CCS diagnosed with CNS tumors from 1958 to 1990, the household income distribution was skewed towards the lower income quartiles (Q4, highest: 15.6%, Q3: 20.6%, Q2: 24.1%, Q1, lowest: 39.6%). The same skew towards the lower income quartiles was not as evident among long-term CCS diagnosed with other cancer types during the same time period (1958–1990), nor among long-term CCS diagnosed with CNS tumors in later years (1991–2018). Further examination of the income distribution of all adult CNS tumor survivors by attained age, as opposed to by year of diagnosis, suggested that lower income was characteristic of all CNS tumor survivors, with the exception of young adults ages 19–25 years (Online Resource Table 18).

### Temporal and projected prevalence

**Fig. 3:**
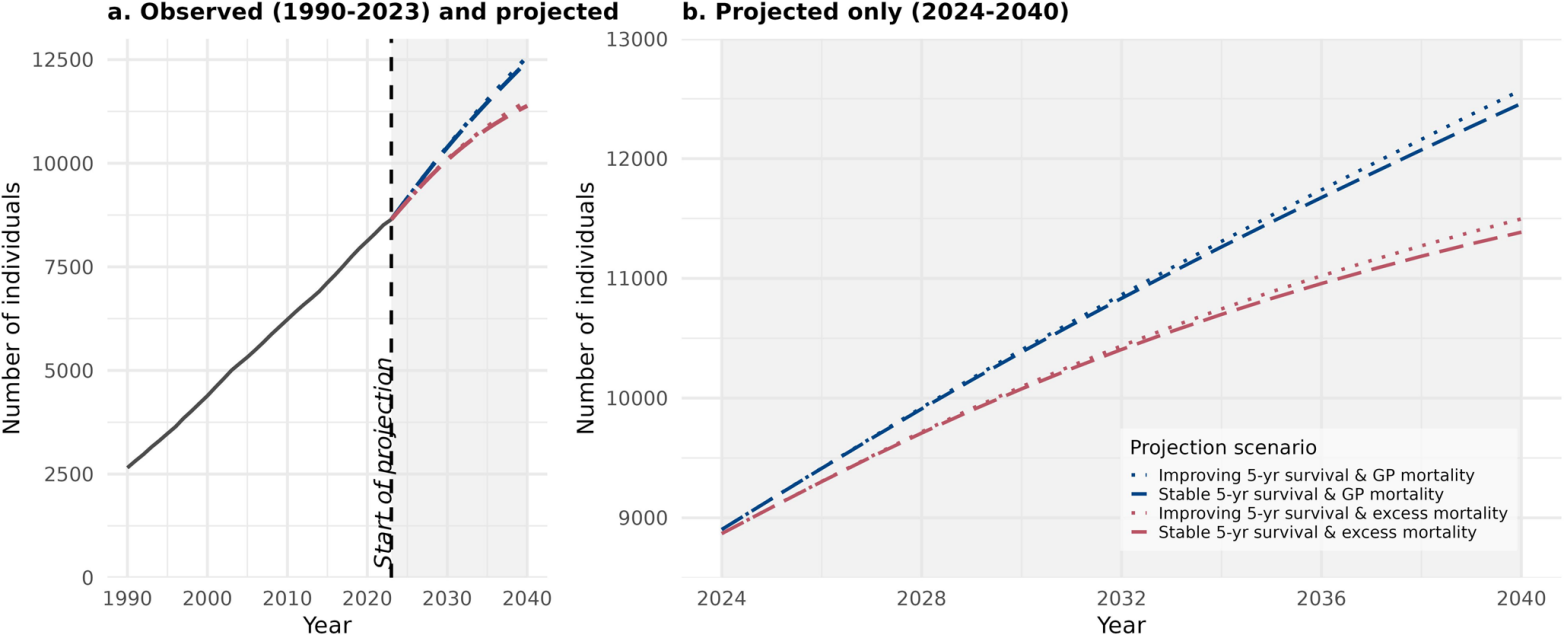
**a** Observed (1990–2023) and projected (2024–2040) prevalence of the number of long-term childhood cancer survivors (diagnosed at ages 0–14 years, from 1958–2018, ≥ 5 years post-diagnosis) registered as living in Sweden. **b** Projected prevalence of the number of long-term childhood cancer survivors living in Sweden under four alternative scenarios. Dashed lines assume short-term (5-year) survival after a childhood cancer diagnosis will remain stable at recent reported levels (in Sweden). Dotted lines assume survival will continue to improve at the rate for individuals diagnosed in Sweden from 1990–2018. Blue lines indicate that childhood cancer survivors have the same long-term (≥ 5 years post-diagnosis) mortality rate as individuals of the same age and sex in the general Swedish population, whereas pink lines assume childhood cancer survivors experience excess mortality compared to the general population at similar rates that have been previously reported. GP = general population. All figures were generated using R version 4.3.1

As illustrated in Fig. 3, the population of long-term CCS more than tripled in size between 1990 and 2023, corresponding to a mean annual percent increase of 3.7% (Online Resource Table 19). Building on this trend, projections indicated continued growth of the long-term CCS population through 2040 (Fig. 3), with a mean annual percent increase between 1.6% and 2.2% (Online Resource Table 20). In the most liberal projection (assuming improving short-term survival and general population mortality rates), there will be an estimated 12,600 long-term CCS in 2040. In the most conservative projection (assuming stable short-term survival and excess long-term mortality), there will be an estimated 11,400 long-term CCS living in Sweden in 2040. Projections by attained age suggested that growth was concentrated among adults (aged > 18 years) (Online Resource Table 21, Online Resource Fig. 2).

## Discussion

There were 8645 long-term CCS (diagnosed ages 0–14 years) living in Sweden in 2023, equivalent to 921 per million persons. The long-term CCS population was heterogeneous. While long-term CCS previously diagnosed with leukemias and CNS tumors were most common, the relative frequency of cancer types differed between younger and older survivors. Approximately 25–30% of long-term CCS had no recent diagnoses or prescriptions, while a similar proportion experienced substantial morbidity. The most prevalent group of diseases diagnosed in the preceding five years were diseases of the eyes and ears. Substantial portions of the adult long-term CCS population were employed, had attained a university education, and had formed families. Considering trends over time, the population of long-term CCS living in Sweden more than tripled in size from 1990 to 2023 and was projected to grow to between 11,400 − 12,600 long-term CCS in 2040.

This study described long-term CCS prevalence in Sweden in 2023, where the last prevalence estimate in the academic literature is over 40 years old [33]. Given this lack of evidence, stakeholders have largely relied on grey literature [34–36], where recent approximations range from 11,000 to 12,000 CCS in Sweden [36, 37]. Discrepancies between the grey literature and our estimate likely reflect varying definitions of age at diagnosis, inclusion of short-term survivors, and use of more limited historical data sources. Our study provides a transparent, accessible estimate of the number of long-term CCS living in Sweden. This figure will be essential for planning survivorship care at the national level [10], for instance, in organizing screening for late effects and ensuring specialized survivorship clinics are adequately staffed.

Our research suggests that approximately 1 in 1000 Swedes is a long-term CCS. Prevalence proportions were highest among adolescents and young adults (aged 15–29 years) and declined across the lifespan. This pattern reflects low childhood cancer survival rates during the 1950–1960s, followed by relatively stable, high survival since the 1990s [2, 14]. Consistent with our results, previous studies from high-income countries investigating CCS diagnosed at ages 0–14 years have reported similar prevalence (651–1101 per million) [12, 13, 16, 17, 38]. Thus, our estimates are likely generalizable to other high-income countries with similar survival rates.

From a clinical and sociodemographic perspective, the long-term CCS population is markedly heterogeneous. Notably, we observed striking differences in the distribution of cancer types across age subsets of the long-term CCS population, reflecting historical differences in treatment advancements [14, 15]. Our study found, for example, that while survivors of leukemias made up a large proportion of younger CCS, there were very few survivors of leukemias over the age of 55. Leukemias were essentially fatal before dramatic improvements in treatment since the 1960s [14, 15]; today the large majority of children diagnosed with leukemias in Europe survive at least five years from their diagnosis [2, 14]. There was also significant heterogeneity with respect to recent disease burden. Approximately 25–30% of long-term CCS had no recent diagnoses in inpatient or outpatient specialist care nor any recent prescriptions, while a similar proportion experienced substantial morbidity. This finding supports evidence from the United Kingdom that some survivors require little follow-up and others have substantial care needs [39], reinforcing the potential of risk-stratified care across European contexts. We also found that survivors of CNS tumors disproportionately lived in lower-income households. Previous studies have identified CNS tumor survivors as socioeconomically vulnerable [7, 40], but have not distinguished by year of diagnosis or attained age. Our study suggests that financial vulnerability is concentrated among survivors of CNS tumors ages 26 and above. The relatively high household income among CNS tumor survivors ages 19–25, however, likely reflects continued residence with their parents. As a result, their underlying financial vulnerability may be obscured. Altogether, our study highlights population-level heterogeneity that is difficult to capture in etiological studies but crucial to consider when designing interventions for long-term CCS.

In line with studies from other contexts, we found that the long-term CCS population in Sweden grew substantially in recent decades, with an annual percent increase of 3.7% from 1990 to 2023. This estimate is slightly higher than in most other studies (around 2.0%) [12, 13, 16]. This is likely explained by differences in how prevalence was defined. Our measure captured all long-term CCS diagnosed since 1958, but not individuals diagnosed before this. Our increase therefore partly reflects an increase in coverage of prevalent long-term CCS over time. Nonetheless, many previous studies are limited to reporting five- or ten-year prevalence [12, 13]. Our study, providing close to complete prevalence over time, is particularly valuable because late effects of cancer treatment and the need for follow-up care often extend beyond ten years after diagnosis.

Building on the limited evidence available from North America [15, 18], our study projected continued growth of the long-term CCS population through 2040 across four different scenarios. Notably, the magnitude of increase was more sensitive to assumptions about long-term mortality than short-term survival, likely because short-term survival rates in Sweden are already high [14]. If ongoing efforts within pediatric oncology, supportive care, and long-term follow-up care succeed in reducing late morbidity, future prevalence will likely be between the estimates based on historical excess mortality and those assuming general population mortality. Our projections highlight the importance of reducing long-term mortality among existing long-term CCS to sustain population growth in the decades ahead, an insight relevant to settings where short-term survival is already high.

Some limitations should be noted. CCS diagnosed abroad or before 1958 were not included in prevalence estimates. On one hand, few survivors treated pre-1958 would be expected to live until 2023, given the intensive therapies and poor survival characteristic of that era [14, 15]. On the other hand, the number of foreign-born people living in Sweden has grown substantially since 2000, amounting to roughly 20% of the Swedish population in 2023 [41]. Thus, our estimate is likely missing a number of hard-to-reach long-term CCS who were diagnosed with childhood cancer abroad and subsequently moved to Sweden. Quantifying this underestimation is challenging. The net emigration rate in our population of long-term CCS was low; but, to our knowledge, the migration patterns of childhood cancer survivors have not been studied. Given that this group of long-term CCS may also benefit from survivorship care, future studies in this area are needed. Additionally, our clinical description did not include treatment data and our disease burden analyses were limited to outpatient specialist and inpatient diagnoses. Thus, conditions managed in primary care or those likely to go undiagnosed are underrepresented in disease burden analyses. Nevertheless, our description of prescribed drug use provided some insight into patterns of primary care utilization.

The strengths of this study stem from the use of nationwide population-based registers. Our study included all long-term CCS diagnosed in Sweden since 1958 and followed them through 2023 with virtually no loss to follow-up. To our knowledge, these are the most comprehensive estimates of long-term CCS prevalence published to date, providing an important benchmark for stakeholders relying on more limited cancer registers. This study’s up-to-date prevalence estimates and detailed descriptions across clinical and sociodemographic subgroups, focused specifically on long-term CCS, provide evidence directly relevant for planning survivorship care. Our broader description serves as a practical resource for clinicians, policymakers, CCS, and their families to better understand the current state of survivorship, a need that will only intensify as this population continues to grow in the coming decades.

## Supplementary Information

Below is the link to the electronic supplementary material.

Supplementary Material 1
