# Supplementary material for: Prevalence, characteristics, and projection of long-term childhood cancer survivors in Sweden
Source: Eur J Epidemiol. 2026 May 7;41(6):775–87. doi: 10.1007/s10654-026-01394-2 (PMC13423994; doi:10.1007/s10654-026-01394-2)
Supplement: Supplementary file 1 — Supplementary Material 1 [file 10654_2026_1394_MOESM1_ESM.docx]

**Online Resources**

*Prevalence, characteristics, and projection of long-term childhood cancer survivors in Sweden*

European Journal of Epidemiology

Elena Extrand^1^; Emerald G Heiland^1^; Genevieve Allen^1^; Mia Giertz^2^; Hanna Mogensen*^3^; Hannah L Brooke*^1^

1 Medical Epidemiology, Department of Surgical Sciences, Uppsala University, Uppsala, Sweden

2 Department of Women and Children’s Health, Uppsala University, Uppsala, Sweden

3 Department of Immunology, Genetics and Pathology, Cancer Precision Medicine, Uppsala University, Uppsala, Sweden

*Denotes equal contribution and shared last authorship

Corresponding author: Hannah L Brooke, [hannah.brooke@uu.se](mailto:hannah.brooke@uu.se)

Online Resources

**Online Resource Figure 1**. Participant flow diagram illustrating selection of long-term childhood cancer survivors living in Sweden on December 31^st^, 2023

**Online Resource Methods 1**. Classifying tumor malignancy and type

**Online Resource Methods 2**. Disease burden analyses, ICD-10 codes

**Online Resource Methods 3.** Prevalence projections, extended methodology

**Online Resource Methods 4.** Modelled standardized mortality ratios (SMRs) for projected long-term mortality

**Online Resource Table 1**. Prevalence of long-term childhood cancer survivors in Sweden on December 31^st^, 2023.

**Online Resource Table 2**. Prevalence (per million persons) of long-term childhood cancer survivors living in Sweden on December 31^st^, 2023.

**Online Resource Table 3**. Basic characteristics of long-term childhood cancer survivors living in Sweden on December 31^st^, 2023

**Online Resource Table 4.** Burden of infectious disease in the last 5 years among long-term childhood cancer survivors

**Online Resource Table 5.** Burden of blood disease in the last 5 years among long-term childhood cancer survivors

**Online Resource Table 6.** Burden of endocrine and other metabolic disease in the last 5 years among long-term childhood cancer survivors

**Online Resource Table 7.** Burden of mental and behavioral disorders in the last 5 years among long-term childhood cancer survivors

**Online Resource Table 8.** Burden of nervous system diseases in the last 5 years among long-term childhood cancer survivors

**Online Resource Table 9.** Burden of diseases of the eyes and ears in the last 5 years among long-term childhood cancer survivors

**Online Resource Table 10.** Burden of circulatory disease in the last 5 years among long-term childhood cancer survivors

**Online Resource Table 11.** Burden of respiratory disease in the last 5 years among long-term childhood cancer survivors

**Online Resource Table 12.** Burden of digestive disease in the last 5 years among long-term childhood cancer survivors

**Online Resource Table 13.** Burden of urinary and genital diseases in the last 5 years among long-term childhood cancer survivors

**Online Resource Table 14.** Burden of skin diseases in the last 5 years among long-term childhood cancer survivors

**Online Resource Table 15.** Burden of bone, joint, and muscle diseases in the last 5 years among long-term childhood cancer survivors

**Online Resource Table 16**. Recent disease burden among long-term childhood cancer survivors diagnosed between 1958 and 1990, by cancer type

**Online Resource Table 17**. Recent disease burden among long-term childhood cancer survivors diagnosed between 1991 and 2018, by cancer type

**Online Resource Table 18.** Household income distribution of long-term central nervous system tumor survivors, by attained age in 2023

**Online Resource Table 19.** Prevalence of long-term childhood cancer survivors in Sweden between 1990-2023.

**Online Resource Table 20.** Projected prevalence of long-term childhood cancer survivors living in Sweden, 2024-2040, across different scenarios

**Online Resource Table 21**. Projected prevalence of long-term childhood cancer survivors living in Sweden by attained age, 2024-2040 (Scenarios 2 and 3)

**Online Resource Figure 2.** Projected prevalence of long-term childhood cancer survivors living in Sweden by attained age, 2024-2040, assuming improving (5-year) mortality and long-term mortality rates of the general Swedish population

Online Resource References

**Online Resource Figure 1**: Participant flow diagram illustrating selection of long-term childhood cancer survivors living in Sweden on December 31^st^, 2023


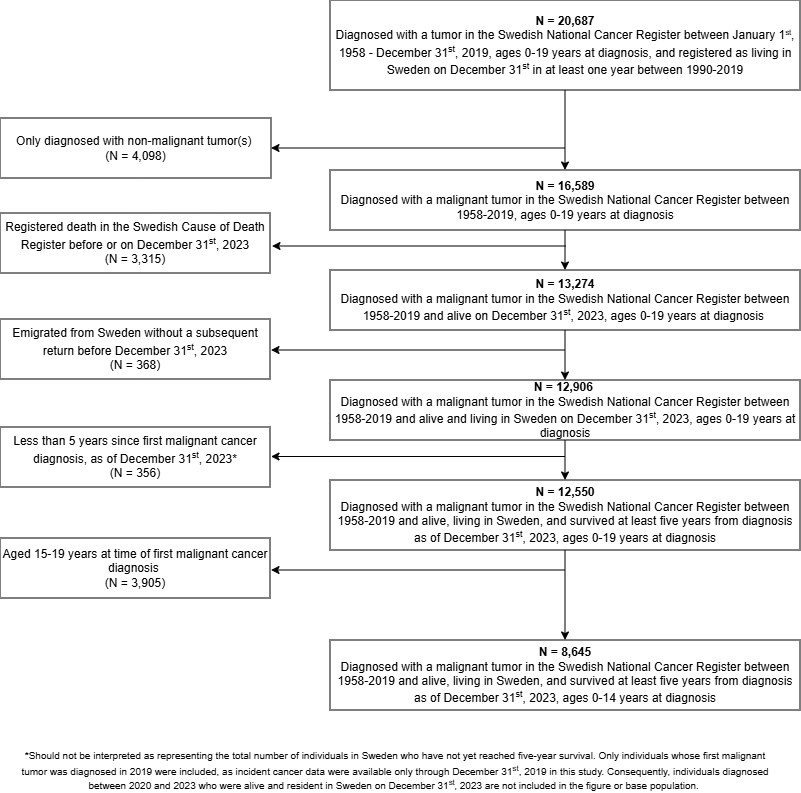


**Online Resource Methods 1**. Classifying tumor malignancy

Morphology and topography codes were used to classify childhood cancer into 12 groups according to the International Classification of Childhood Cancer, 3rd edition, 2017 update (ICCC-3-2017).^1,2^ Malignancy was determined using behavior codes contained in the National Cancer Register.^3^ Leukemias (groups Ia-Ie), central nervous system tumors (groups IIIa-IIIf), and intracranial and intraspinal germ cell tumors (group Xa) were considered malignant by site and included regardless of tumor morphology. Individuals diagnosed with lymphomas (groups IIa, IIc-IIe), with the exception of non-Hodgkin lymphomas (group IIb), were also included if their tumor’s morphology code indicated suspicion of a malignant tumor.

**Online Resource Methods 2**. Disease burden analyses, ICD-10 codes

The following ICD-10 codes were used to define disease groups and sub-groups used in the disease burden analyses. Classifications were primarily based on previous Scandinavian cohort studies exploring health outcomes among long-term childhood cancer survivors.^4,5^ Diagnoses were extracted from the National Patient Register and included primary and secondary diagnoses (positions 1-30) from outpatient specialist care and inpatient care but not primary care.^6^

Only diagnoses in the 5-year period preceding December 31^st^, 2023 were included in disease burden analyses. To avoid capturing acute complications of cancer treatment (i.e., among those diagnosed in more recent years, 2016-2018), diagnoses that occurred <2 years after cancer diagnosis were excluded.

**Online Resource Methods 2 Table 1**. ICD-10 codes used in disease burden analyses to define disease groups and sub-groups.

| Disease Group | ICD-10 Codes |
| --- | --- |
| **Infectious and parasitic diseases** |  |
| Intestinal infectious diseases | A00–A09 |
| Tuberculosis | A15–A19 |
| Sepsis | A40–A41 |
| Erysipelas | A46 |
| Other bacterial diseases | A30–A39, A42–A45, A47–A49 |
| Enterovirus diseases of CNS | A80–A81, A83–A89 |
| Herpes zoster | B02 |
| Other viral diseases with exanthem | B00–B01, B03–B09 |
| Infectious hepatitis, HIV infection (only in ICD-9 and -10) and other viral diseases | A82, B15–B34 |
| Syphilis and other venereal diseases | A50–A71, A74 |
| Mycoses | B35–B49 |
| Other infectious and parasitic diseases | A20–A28, A75–A79, A90–A99, B50–B83, B85–B99 |
| **Benign tumors** | D00–D05, D07–D08, D09.2–D30.0, D31, D34–D35.1, D35.5–D36 |
| **Diseases of the blood and blood-forming organs** |  |
| Anemias | D50–D54, D59–D64 |
| Coagulation defects, purpura and other hemorrhagic conditions | D65–D69 |
| Agranulocytosis | D70–D72 |
| Other diseases of the blood and blood-forming organs | D73–D79, D86, D89 |
| **Endocrine diseases, nutritional deficiencies and other metabolic diseases** |  |
| Diseases of the thyroid gland | E01–E02, E03.2–E07 |
| Diabetes mellitus | E10–E14 |
| Other disorders of glucose regulation and pancreatic internal secretion | E15-E16 |
| Pituitary hypofunction | E23.0–E23.3 |
| Ovarian dysfunction | E28 |
| Testicular dysfunction | E29 |
| Disorders of other endocrine organs | E20–E22, E23.6–E27, E30–E35 |
| Nutritional deficiencies | E40–E64 |
| Obesity | E65-68 |
| Other metabolic disorders | E73, E86–E90 |
| **Mental and behavioral disorders** |  |
| Organic psychoses | F06.0-F06.3 |
| Schizophrenia and other, non-affective psychoses | F20-F29 |
| Affective disorders | F30-F39 |
| Anxiety, obsessive compulsive disorders, and dissociative disorders | F40-F42, F44, F48.1-F48.9 |
| Stress-related disorders | F43 |
| Disorders of adult personality and behavior | F60-F69 |
| Attention deficit hyperactivity disorders (ADHD) | F90, F98.8 |
| Autism spectrum disorder | F84 |
| Disorders related to psychoactive substance use | F10-F19 |
| Developmental disorders  and intellectual  disability | F70-F79, F80-F83, F88-89, F98.5, F98.6 |
| Organic, including  symptomatic, mental  disorders | F00-03, F04, F05, F06.4-F06.9, F07, F09 |
| Somatoform and other  neurotic disorders | F45, F48.0 |
| Other emotional and  behavioral disorders | F50-F55, F59, F91-F94, F95, F98.0-F98.4, F98.9, F99 |
| **Diseases of the nervous system** |  |
| Meningitis | G00–G03 |
| Other inflammatory diseases of CNS | G04–G09 |
| Multiple sclerosis and other demyelinating diseases of CNS | G35–G37 |
| Parkinson disease and other movement disorders | G20–G22, G24–G26 |
| Epilepsy | G40–G41 |
| Migraine and other diseases of the brain and spinal cord | G13, G43–G44, G46–G47 |
| Senile and pre-senile dementia | G30–G32 |
| Diseases of the nerves and peripheral ganglia | G50–G59, G61–G73, G81–G90.0, G90.2–G99 |
| **Diseases of the eyes and ears** |  |
| Inflammatory and other diseases of the eye | H00–H22, H30–H36, H43–H59 |
| Cataract | H25–H28, H40–H42 |
| Inflammatory diseases of the ear | H60–H75 |
| Ménière's disease and otosclerosis | H80–H82 |
| Other diseases of the ear and deafness | H83–H95 |
| **Diseases of the circulatory system** |  |
| Acute rheumatic fever | I00–I02 |
| Chronic rheumatic heart disease | I05–I09 |
| Hypertensive disease | I10–I15 |
| Ischemic heart disease | I20–I25 |
| Pulmonary heart disease | I26–I28 |
| Pericardial–, myocardial–, and endocardial disease | I30–I33, I38–I41, I51.4 |
| Valvular disease (non-rheumatic) | I34–I37 |
| Heart failure | I42–I43, I50, I51.5, I51.7 |
| Conduction disorders | I44–I49 |
| Cerebrovascular disease | I60–I69, G45 |
| Diseases of arteries, arterioles, and capillaries | I70–I79 |
| Venous and lymphatic disease | I80–I89 |
| Other complications of the circulatory system | I51.0–I51.3, I51.6, I51.8–I51.9, I52, I95–I99, M30 |
| **Diseases of the respiratory system** |  |
| Influenza | J10–J11 |
| Acute upper respiratory infections | J00–J06 |
| Other disorders of the upper respiratory tract | J30–J39 |
| Pneumonia | J12–J18 |
| Abscess of lung and pyothorax | J85–J86, J90 |
| Bronchitis and emphysema | J20–J22, J40–J44, J47, J98.2–J98.3 |
| Asthma | J45–J46 |
| Lung diseases due to external agents | J60–J70 |
| Interstitial pulmonary diseases and pulmonary oedema | J81–J84 |
| Pneumothorax | J93 |
| Respiratory failure | J96, J98.1 |
| Other diseases of respiratory system | J80, J91–J92, J94–J95, J98.0, J98.4–J98.9, J99 |
| **Diseases of the digestive organs** |  |
| Diseases of the teeth and supporting structures | K00–K08 |
| Other diseases of the oral cavity and salivary glands | K09–K14 |
| Diseases of the esophagus | K20–K23 |
| Diseases of the stomach and duodenum | K25–K31 |
| Appendicitis | K35–K38 |
| Hernia of the abdominal cavity | K40–K46 |
| Non-infective enteritis and colitis | K50–K52 |
| Paralytic ileus and intestinal obstruction | K56 |
| Diseases of the anal and rectal regions | K60–K62 |
| Diseases of the peritoneum | K65–K67 |
| Other diseases of the digestive system | K55, K57–K59, K63, K90–K93 |
| Diseases of the liver | K70–K77 |
| Diseases of the gallbladder and biliary ducts | K80–K83, K87 |
| Diseases of the pancreas | K85–K86 |
| **Diseases of the urinary system and genital organs (including infertility)** |  |
| Glomerular Disease | N00–N01, N03–N05, N02.0–N02.8, N06.0–N06.8, N07.0–N07.8, N08 |
| Acute renal failure | N17, N28.0 |
| Chronic kidney disease | N11.8–N11.9, N12, N18–N19, N26–N27, N28.1 |
| Urolithiasis | N20–N22 |
| Obstructive uropathy | N11.0–N11.1, N13.0–N13.5, N13.8–N13.9, N32.0, N35 |
| Infections of the urinary system | N10, N13.6, N15.1, N16.0, N29.0–N29.1, N30, N33.0, N34, N37.0, N39.0 |
| Other an unspecified disorders of the urinary system | N02.9, N06.9, N07.9, N13.7, N14, N15.0, N15.8–N15.9, N16.1– N16.8, N23, N25, N28.8–N28.9, N29.8, N31, N32.1–N32.9, N33.8, N36, N37.8, N39.1, N39.2–N39.9 |
| Diseases of the prostate | N40–N42 |
| Hydrocoele and spermatocoele | N43 |
| Orchitis and epididymitis | N45 |
| Other diseases of the male genital organs | N44, N47–N51 |
| Chronic cystic disease and other diseases of the breast | N60–N64 |
| Inflammatory diseases of the female pelvic organs | N70–N77 |
| Endometriosis | N80 |
| Noninflammatory disorders of the female genital tract | N81–N90 |
| Male sterility | N46 |
| Abnormal menstruation | N91–N92 |
| Female infertility | N97 |
| Other disorders of the female reproductive system | N93–N96, N98 |
| **Diseases of the skin and subcutaneous tissues** |  |
| Infections of the skin and subcutaneous tissue | L00–L08 |
| Other inflammatory conditions of the skin and subcutaneous tissue | L10–L57, L59 |
| Radiodermatitis | L58 |
| Disorders of skin appendages (hair, nails, sweat glands) | L60–L75 |
| Other disorders of the skin and subcutaneous tissue | L80–L99 |
| **Diseases of bones, joints, and muscles** |  |
| Arthritis and rheumatism | M00–M19, M79.0 |
| Osteomyelitis and other diseases of bone and joints | M20–M25, M40–M54, M80–M94 |
| Other diseases of the musculoskeletal system | M31–M36, M60–M77, M79.1–M79.9, M95–M99 |

**Online Resource Methods 3.** Prevalence projections, extended methodology

The absolute prevalence of long-term childhood cancer survivors (CCS) was projected from 2024 to 2040 by simulating the inflow and outflow of individuals from the existing observed population on December 31^st^, 2023.

**Inflow**

The inflow, or number of incident long-term CCS in a given year, was measured by incorporating information on the size of the underlying population at risk, the incidence rate of childhood cancer, and the 5-year (short-term) mortality rate among children diagnosed with cancer. All inflow data were obtained stratified by age (0-4, 5-9, 10-14) and sex (male/female) and accounted for the 5-year lag between diagnosis and entering the population as an incident long-term CCS.

First, to obtain the size of the underlying population at risk, the number of children registered as living in Sweden on December 31^st^ five years prior to the projection year was obtained from Statistics Sweden online databases. Both actual (2019-2024) and projected (2025-2035) population sizes were included. Next, incidence rates of childhood cancer from 2008-2018 were extracted from the National Cancer Register’s statistical tool.^7^ These rates were averaged and applied uniformly over the projection period under the assumption that childhood cancer incidence will remain relatively stable. Finally, two distinct scenarios of short-term survival were incorporated. Both scenarios integrate mean 5-year mortality rates from 2013-2023, as determined using individual-level data from the National Cancer Register and the Cause of Death Register. In the first scenario, it is assumed that the 5-year mortality rate observed from 2013-2023 will persist until 2040. In the second scenario, it is assumed that 5-year survival rates will increase steadily across the projection period. The degree of increase was derived using observed 5-year survival rates between 1990 and 2018. The annual absolute increase in the 5-year survival rate was assessed using logistic regression with year of diagnosis (1990–2018) as the predictor, indicating an average increase of 0.28 percentage points per year.

**Outflow**

The outflow, or number of prevalent long-term CCS who exit or re-enter the population in a given year, was quantified using data on migration and long-term mortality.

The net migration rate accounted for both emigration and re-immigration. Observed net migration rates of long-term CCS between 2013 and 2023 were estimated using migration data from the Total Population Register. The mean annual rate during this time period was a loss of 0.78 persons per 1,000 individuals. This rate was then applied to the period from 2024-2040 and assumed to remain constant.

In parallel, projected mortality rates for the general Swedish population (2024–2040) were obtained from Statistics Sweden’s online database in 1-year age intervals by sex.^8^ These mortality rates formed the basis of two distinct long-term mortality (mortality >5 years after diagnosis) scenarios. The first assumed long-term CCS would have the same mortality rate as age- and sex- matched members of the general Swedish population. By contrast, the second scenario assumed CCS experience excess mortality compared to the general population. The magnitude of excess, by years since diagnosis, was determined using standardized mortality ratios (SMRs) from a meta-analysis of mortality among long-term CCS.^9^ To model the excess long-term mortality scenario, standardized mortality ratios (SMRs) by years since diagnosis were estimated using random-effects meta-regression with restricted cubic splines (3 degrees of freedom). SMRs were log-transformed, and standard errors were derived from reported confidence intervals. Point estimates were predicted for each year from 5-60 years after diagnosis. Modeled SMR values are available in Online Resource Methods 4. Since the review did not include SMR data beyond 60 years post-diagnosis, SMRs were assumed to remain constant thereafter.

**Projection Scenarios**

Four discrete projection scenarios were modeled, all of which assumes incidence would be stable across the study period. The four distinct projection scenarios are summarized below.

1. Scenario 1: Incidence is assumed to remain stable at the age- and sex-specific mean rates observed in Sweden during 2008–2018. Five-year survival is likewise assumed to remain stable at the age- and sex-specific mean rates observed in 2013–2023 among individuals diagnosed between 2008–2018. Long-term mortality is projected to be equal to that of age- and sex-matched members of the Swedish general population.
2. Scenario 2: Incidence is assumed to remain stable at the age- and sex-specific mean rates observed in Sweden during 2008–2018. Five-year survival is likewise assumed to remain stable at the age- and sex-specific mean rates observed in 2013–2023 among individuals diagnosed between 2008–2018. Long-term mortality is projected to reflect excess mortality compared with age- and sex-matched members of the general population, varying according to years since diagnosis.
3. Scenario 3: Incidence is assumed to remain stable at the age- and sex-specific mean rates observed in Sweden during 2008–2018. Five-year survival is projected to steadily improve, with an estimated annual increase of 0.28% in the age- and sex-specific survival rates. Long-term mortality is projected to be equal to that of age- and sex-matched members of the Swedish general population.
4. Scenario 4: Incidence is assumed to remain stable at the age- and sex-specific mean rates observed in Sweden during 2013–2023. Five-year survival is projected to steadily improve, with an estimated annual increase of 0.28% in the age- and sex-specific survival rates. Long-term mortality is projected to reflect excess mortality compared with age- and sex-matched members of the general population, varying according to years since diagnosis.

**Online Resource Methods 4**. Statistical Analyses for projections, including modelled standardized mortality ratios for projected long-term mortality

Prevalence beyond December 31^st^, 2023, was estimated using an iterative, stepwise projection model based on annual inflow and outflow of long-term CCS in Sweden. Projections were also generated stratified by age at the end of the projected year (5-18 years, 19-35 years, 36-55 years, and >55 years). Inflow was modeled by obtaining the product of the underlying population at risk, the incidence rate of childhood cancer, and the short-term mortality rate within each stratum of age and sex. Similarly, outflow was estimated by multiplying the prior year’s population by the migration and long-term mortality rate. One of the two long-term mortality scenarios presumed excess mortality, in which case the long-term mortality rate was multiplied by modelled standardized mortality ratios (SMR). Excess long-term mortality was modeled using previously published standardized mortality ratios,^9^ estimated by years since diagnosis via random-effects meta-regression with restricted cubic splines. Standardized mortality ratios (SMRs) were extracted from Moskalewicz et al.’s systematic review and meta-analysis of late mortality among childhood cancer survivors (2024).^9^ Only the non-overlapping studies included in their meta-analysis were plotted over time.^10–18^

In the table below, the subsequent predicted SMRs used to inform assumptions about excess mortality in this study and their corresponding confidence intervals are listed by year since diagnosis. For those who were more than 60 years since their cancer diagnosis, the SMR for those at 60 years since diagnosis was assumed to apply (SMR: 1.66).

**Online Resource Methods 4 Table 1:** Predicted standardized mortality ratios by years since diagnosis and corresponding confidence intervals.

| Years since diagnosis | Predicted SMR | Lower CI | Upper CI | Years since diagnosis | Predicted SMR | Lower CI | Upper CI |
| --- | --- | --- | --- | --- | --- | --- | --- |
| 5 | 27.46 | 22.46 | 33.56 | 33 | 5.3 | 4.51 | 6.22 |
| 6 | 24.45 | 20.3 | 29.46 | 34 | 5.36 | 4.53 | 6.35 |
| 7 | 21.79 | 18.33 | 25.89 | 35 | 5.43 | 4.55 | 6.48 |
| 8 | 19.43 | 16.55 | 22.82 | 36 | 5.49 | 4.56 | 6.6 |
| 9 | 17.36 | 14.93 | 20.17 | 37 | 5.53 | 4.57 | 6.7 |
| 10 | 15.53 | 13.48 | 17.9 | 38 | 5.56 | 4.57 | 6.77 |
| 11 | 13.94 | 12.17 | 15.95 | 39 | 5.57 | 4.55 | 6.81 |
| 12 | 12.54 | 11 | 14.29 | 40 | 5.54 | 4.52 | 6.8 |
| 13 | 11.32 | 9.96 | 12.88 | 41 | 5.48 | 4.46 | 6.74 |
| 14 | 10.27 | 9.03 | 11.67 | 42 | 5.39 | 4.39 | 6.61 |
| 15 | 9.35 | 8.22 | 10.63 | 43 | 5.26 | 4.3 | 6.43 |
| 16 | 8.56 | 7.51 | 9.75 | 44 | 5.09 | 4.18 | 6.21 |
| 17 | 7.87 | 6.89 | 8.99 | 45 | 4.9 | 4.04 | 5.94 |
| 18 | 7.29 | 6.37 | 8.34 | 46 | 4.69 | 3.88 | 5.66 |
| 19 | 6.79 | 5.92 | 7.79 | 47 | 4.46 | 3.7 | 5.37 |
| 20 | 6.37 | 5.54 | 7.32 | 48 | 4.22 | 3.51 | 5.07 |
| 21 | 6.02 | 5.23 | 6.93 | 49 | 3.97 | 3.29 | 4.79 |
| 22 | 5.73 | 4.98 | 6.6 | 50 | 3.72 | 3.06 | 4.52 |
| 23 | 5.51 | 4.79 | 6.34 | 51 | 3.47 | 2.82 | 4.28 |
| 24 | 5.34 | 4.65 | 6.14 | 52 | 3.23 | 2.57 | 4.05 |
| 25 | 5.22 | 4.55 | 5.99 | 53 | 2.99 | 2.33 | 3.83 |
| 26 | 5.14 | 4.49 | 5.89 | 54 | 2.76 | 2.1 | 3.63 |
| 27 | 5.1 | 4.45 | 5.84 | 55 | 2.55 | 1.88 | 3.45 |
| 28 | 5.08 | 4.44 | 5.83 | 56 | 2.34 | 1.67 | 3.28 |
| 29 | 5.09 | 4.44 | 5.85 | 57 | 2.15 | 1.49 | 3.11 |
| 30 | 5.13 | 4.45 | 5.91 | 58 | 1.97 | 1.31 | 2.96 |
| 31 | 5.17 | 4.47 | 5.99 | 59 | 1.81 | 1.16 | 2.82 |
| 32 | 5.23 | 4.49 | 6.1 | 60 | 1.66 | 1.02 | 2.68 |

**Online Resource Table 1**. Prevalence of long-term childhood cancer survivors in Sweden on December 31^st^, 2023, by age

|  |  | Age (on December 31^st^, 2023) | | | | | | |
| --- | --- | --- | --- | --- | --- | --- | --- | --- |
| Age at diagnosis | Sex | 5-18 | 19-25 | 26-35 | 36-45 | 46-55 | 55+ | All ages |
| Overall (0-14) | Overall | 1705 | 1349 | 2002 | 1518 | 1187 | 884 | 8645 |
|  | Male | 917 | 727 | 1087 | 822 | 619 | 438 | 4610 |
|  | Female | 788 | 622 | 915 | 696 | 568 | 446 | 4035 |
| <1 | Overall | 288 | 121 | 191 | 132 | 106 | 62 | 900 |
|  | Male | 159 | 56 | 92 | 66 | 63 | 35 | 471 |
|  | Female | 129 | 65 | 99 | 66 | 43 | 27 | 429 |
| 1-4 | Overall | 918 | 452 | 745 | 481 | 323 | 218 | 3137 |
|  | Male | 492 | 245 | 398 | 245 | 171 | 103 | 1654 |
|  | Female | 426 | 207 | 347 | 236 | 152 | 115 | 1483 |
| 5-9 | Overall | 403 | 363 | 504 | 439 | 317 | 203 | 2229 |
|  | Male | 217 | 212 | 291 | 254 | 161 | 119 | 1254 |
|  | Female | 186 | 151 | 213 | 185 | 156 | 84 | 975 |
| 10-14 | Overall | 96 | 413 | 562 | 466 | 441 | 401 | 2379 |
|  | Male | 49 | 214 | 306 | 257 | 224 | 181 | 1231 |
|  | Female | 47 | 199 | 256 | 209 | 217 | 220 | 1148 |

Long-term survivors are defined here as individuals diagnosed with cancer between ages 0-14, 1958-2018, who survived >=5 years from diagnosis.

**Online Resource Table 2**. Prevalence proportions (per million persons) of long-term childhood cancer survivors living in Sweden on December 31^st^, 2023.

| Age at diagnosis |  | Age (on December 31^st^, 2023) | | | | | | | | | | | | |  |
| --- | --- | --- | --- | --- | --- | --- | --- | --- | --- | --- | --- | --- | --- | --- | --- |
|  | Sex | 5-9 | 10-14 | 15-19 | 20-24 | 25-29 | 30-34 | 35-39 | 40-44 | 45-49 | 50-54 | 55-59 | 60-64 | 65-79^a^ | Overall |
| Overall (0-14) | Overall | 413 | 1150 | 1529 | 1627 | 1449 | 1352 | 1172 | 1134 | 1018 | 887 | 566 | 424 | 205 | 921 |
|  | Male | 436 | 1205 | 1599 | 1666 | 1500 | 1448 | 1245 | 1195 | 1049 | 897 | 579 | 447 | 192 | 967 |
|  | Female | 388 | 1091 | 1455 | 1584 | 1394 | 1252 | 1095 | 1070 | 986 | 877 | 552 | 401 | 218 | 875 |
| <1 | Overall | 158 | 179 | 157 | 147 | 131 | 138 | 110 | 82 | 85 | 92 | 60 | 34 | 3 | 96 |
|  | Male | 183 | 200 | 136 | 135 | 114 | 135 | 111 | 72 | 101 | 105 | 75 | 37 | 1 | 99 |
|  | Female | 131 | 157 | 179 | 160 | 148 | 142 | 109 | 94 | 70 | 80 | 45 | 31 | 5 | 93 |
| 1-4 | Overall | 255 | 710 | 615 | 556 | 546 | 495 | 397 | 359 | 306 | 210 | 166 | 114 | 35 | 334 |
|  | Male | 253 | 755 | 643 | 576 | 548 | 525 | 399 | 343 | 323 | 227 | 150 | 102 | 38 | 347 |
|  | Female | 258 | 663 | 586 | 534 | 544 | 463 | 394 | 375 | 288 | 193 | 181 | 127 | 31 | 321 |
| 5-9 | Overall | NA | 260 | 508 | 433 | 342 | 346 | 320 | 333 | 279 | 235 | 130 | 85 | 56 | 238 |
|  | Male | NA | 250 | 567 | 476 | 376 | 398 | 378 | 363 | 277 | 236 | 136 | 108 | 68 | 263 |
|  | Female | NA | 271 | 445 | 385 | 306 | 292 | 259 | 300 | 281 | 233 | 125 | 62 | 44 | 211 |
| 10-14 | Overall | NA | NA | 250 | 492 | 429 | 373 | 345 | 360 | 348 | 350 | 210 | 191 | 112 | 254 |
|  | Male | NA | NA | 253 | 479 | 461 | 390 | 356 | 417 | 348 | 329 | 217 | 200 | 84 | 258 |
|  | Female | NA | NA | 246 | 506 | 396 | 355 | 333 | 300 | 348 | 371 | 202 | 182 | 139 | 249 |

a. Prevalence among those ages 65-79 is combined, since these individuals are only partially covered in the current register linkage, which dates back to 1958.

Long-term survivors are defined here as individuals diagnosed with cancer between ages 0-14, 1958-2018, who survived >=5 years from diagnosis. Empty cells or cells marked with 'NA' represent combinations that are not feasible, as survivors must be alive for at least five years post-diagnosis, precluding certain age group pairings. Each cell represents the number of childhood cancer survivors per million persons in the Swedish population of the same age and sex group on December 31^st^, 2023. For example, a value of 544 in the cell corresponding to females diagnosed at ages 1–4 years and aged 25–29 years on December 31^st^, 2023, indicates that there were 544 female survivors who (diagnosed between the ages of 1 and 4) per million Swedish females aged 25–29 years.

**Online Resource Table 3**. Basic characteristics of long-term childhood cancer survivors living in Sweden on December 31^st^, 2023

|  | Minimum | 25^th^ percentile | Median | 75^th^ percentile | Maximum |
| --- | --- | --- | --- | --- | --- |
| Age on December 31^st^ 2023 (years) | 5 | 21 | 32 | 45 | 79 |
| Time since diagnosis (years) | 5 | 14 | 25 | 38 | 65 |
| Age at diagnosis (years) | 0 | 2 | 5 | 10 | 14 |
| Year of diagnosis | 1958 | 1985 | 1998 | 2009 | 2018 |

**Online Resource Table 4**. Burden of infectious disease in the last 5 years among long-term childhood cancer survivors

|  | No. | % |
| --- | --- | --- |
| Intestinal infectious diseases |  |  |
| Yes | 171 | 2.0 |
| No | 8,474 | 98.0 |
| Sepsis |  |  |
| Yes | 67 | 0.8 |
| No | 8,578 | 99.2 |
| Erysipelas |  |  |
| Yes | 58 | 0.7 |
| No | 8,587 | 99.3 |
| Other bacterial diseases |  |  |
| Yes | 70 | 0.8 |
| No | 8,575 | 99.2 |
| Herpes zoster |  |  |
| Yes | 37 | 0.4 |
| No | 8,608 | 99.6 |
| Other viral diseases with exanthem |  |  |
| Yes | 101 | 1.2 |
| No | 8,544 | 98.8 |
| Infectious hepatitis, HIV infection and other viral diseases | | |
| Yes | 261 | 3.0 |
| No | 8,384 | 97.0 |
| Syphilis and other venereal diseases |  |  |
| Yes | 82 | 0.9 |
| No | 8,563 | 99.1 |
| Mycoses |  |  |
| Yes | 152 | 1.8 |
| No | 8,493 | 98.2 |
| Other infectious and parasitic diseases |  |  |
| Yes | 255 | 2.9 |
| No | 8,390 | 97.1 |
| Total | 8,645 | 100.0 |
| To protect confidentiality and minimize disclosure risk, sub-diseases with small cell counts (n ≤ 5) have been omitted from frequency tables. | | |

**Online Resource Table 5**. Burden of blood disease in the last 5 years among long-term childhood cancer survivors

|  | No. | % |
| --- | --- | --- |
| Anaemias |  |  |
| Yes | 247 | 2.9 |
| No | 8,398 | 97.1 |
| Coagulation defects, purpura and other hemorrhagic conditions | | |
| Yes | 107 | 1.2 |
| No | 8,538 | 98.8 |
| Agranulocytosis |  |  |
| Yes | 101 | 1.2 |
| No | 8,544 | 98.8 |
| Other diseases of the blood and blood-forming organs | |  |
| Yes | 56 | 0.6 |
| No | 8,589 | 99.4 |
| Total | 8,645 | 100.0 |

To protect confidentiality and minimize disclosure risk, sub-diseases with small cell counts (n ≤ 5) have been omitted from frequency tables.

**Online Resource Table 6**. Burden of endocrine and other metabolic disease in the last 5 years among long-term childhood cancer survivors

|  |  | No. | % |
| --- | --- | --- | --- |
| Diseases of the thyroid gland |  |  |  |
| Yes |  | 467 | 5.4 |
| No |  | 8,178 | 94.6 |
| Diabetes mellitus |  |  |  |
| Yes |  | 261 | 3.0 |
| No |  | 8,384 | 97.0 |
| Pituitary hypofunction |  |  |  |
| Yes |  | 579 | 6.7 |
| No |  | 8,066 | 93.3 |
| Ovarian dysfunction |  |  |  |
| Yes |  | 155 | 1.8 |
| No |  | 8,490 | 98.2 |
| Testicular dysfunction |  |  |  |
| Yes |  | 110 | 1.3 |
| No |  | 8,535 | 98.7 |
| Disorders of other endocrine organs |  |  |  |
| Yes |  | 401 | 4.6 |
| No |  | 8,244 | 95.4 |
| Nutritional deficiencies |  |  |  |
| Yes |  | 89 | 1.0 |
| No |  | 8,556 | 99.0 |
| Obesity |  |  |  |
| Yes |  | 301 | 3.5 |
| No |  | 8,344 | 96.5 |
| Other metabolic disorders |  |  |  |
| Yes |  | 682 | 7.9 |
| No |  | 7,963 | 92.1 |
| Total |  | 8,645 | 100.0 |

To protect confidentiality and minimize disclosure risk, sub-diseases with small cell counts (n ≤ 5) have been omitted from frequency tables.

**Online Resource Table 7**. Burden of mental and behavioral disorders in the last 5 years among long-term childhood cancer survivors

|  | No. | % |
| --- | --- | --- |
| Organic psychoses |  |  |
| Yes | 7 | 0.1 |
| No | 8,638 | 99.9 |
| Schizophrenia and other, non-affective psychoses | |  |
| Yes | 59 | 0.7 |
| No | 8,586 | 99.3 |
| Affective disorders |  |  |
| Yes | 429 | 5.0 |
| No | 8,216 | 95.0 |
| Anxiety, obsessive compulsive disorders, and dissociative disorders | | |
| Yes | 544 | 6.3 |
| No | 8,101 | 93.7 |
| Stress-related disorders |  |  |
| Yes | 245 | 2.8 |
| No | 8,400 | 97.2 |
| Disorders of adult personality and behavior | |  |
| Yes | 87 | 1.0 |
| No | 8,558 | 99.0 |
| Attention deficit hyperactivity disorders (ADHD) | |  |
| Yes | 493 | 5.7 |
| No | 8,152 | 94.3 |
| Autism spectrum disorder |  |  |
| Yes | 280 | 3.2 |
| No | 8,365 | 96.8 |
| Disorders related to psychoactive substance use | |  |
| Yes | 210 | 2.4 |
| No | 8,435 | 97.6 |
| Developmental disorders and intellectual disability | |  |
| Yes | 311 | 3.6 |
| No | 8,334 | 96.4 |
| Organic, including symptomatic, mental disorders | |  |
| Yes | 174 | 2.0 |
| No | 8,471 | 98.0 |
| Somatoform and other neurotic disorders | |  |
| Yes | 39 | 0.5 |
| No | 8,606 | 99.5 |
| Other emotional and behavioural disorders | |  |
| Yes | 231 | 2.7 |
| No | 8,414 | 97.3 |
| Total | 8,645 | 100.0 |

To protect confidentiality and minimize disclosure risk, sub-diseases with small cell counts (n ≤ 5) have been omitted from frequency tables.

**Online Resource Table 8**. Burden of nervous system diseases in the last 5 years among long-term childhood cancer survivors

|  | No. | % |
| --- | --- | --- |
| Meningitis |  |  |
| Yes | 12 | 0.1 |
| No | 8,633 | 99.9 |
| Other inflammatory diseases of CNS |  |  |
| Yes | 17 | 0.2 |
| No | 8,628 | 99.8 |
| Multiple sclerosis and other demyelinating diseases of CNS | | |
| Yes | 18 | 0.2 |
| No | 8,627 | 99.8 |
| Parkinson disease and other movement disorders | |  |
| Yes | 40 | 0.5 |
| No | 8,605 | 99.5 |
| Epilepsy |  |  |
| Yes | 520 | 6.0 |
| No | 8,125 | 94.0 |
| Migraine and other diseases of the brain and spinal cord | | |
| Yes | 454 | 5.3 |
| No | 8,191 | 94.7 |
| Senile and pre-senile dementia |  |  |
| Yes | 8 | 0.1 |
| No | 8,637 | 99.9 |
| Diseases of the nerves and peripheral ganglia | |  |
| Yes | 629 | 7.3 |
| No | 8,016 | 92.7 |
| Total | 8,645 | 100.0 |

To protect confidentiality and minimize disclosure risk, sub-diseases with small cell counts (n ≤ 5) have been omitted from frequency tables.

**Online Resource Table 9**. Burden of diseases of the eyes and ears in the last 5 years among long-term childhood cancer survivors

|  | No. | % |  |
| --- | --- | --- | --- |
| Inflammatory and other diseases of the eye | |  |  |
| Yes | 1,443 | 16.7 |  |
| No | 7,202 | 83.3 |  |
| Cataract |  |  |  |
| Yes | 299 | 3.5 |  |
| No | 8,346 | 96.5 |  |
| Inflammatory diseases of the ear |  |  |  |
| Yes | 419 | 4.8 |  |
| No | 8,226 | 95.2 |  |
| Ménière's disease and otosclerosis |  |  |  |
| Yes | 89 | 1.0 |  |
| No | 8,556 | 99.0 |  |
| Other diseases of the ear and deafness |  |  |  |
| Yes | 506 | 5.9 |  |
| No | 8,139 | 94.1 |  |
| Total | 8,645 | 100.0 |  |
| To protect confidentiality and minimize disclosure risk, sub-diseases with small cell counts (n ≤ 5) have been omitted from frequency tables. | | | |

**Online Resource Table 10**. Burden of circulatory disease in the last 5 years among long-term childhood cancer survivors

|  | No. | % |
| --- | --- | --- |
| Chronic rheumatic heart disease |  |  |
| Yes | 15 | 0.2 |
| No | 8,630 | 99.8 |
| Hypertensive disease |  |  |
| Yes | 451 | 5.2 |
| No | 8,194 | 94.8 |
| Ischemic heart disease |  |  |
| Yes | 70 | 0.8 |
| No | 8,575 | 99.2 |
| Pulmonary heart disease |  |  |
| Yes | 46 | 0.5 |
| No | 8,599 | 99.5 |
| Pericardial, myocardial, and endocardial disease | |  |
| Yes | 30 | 0.3 |
| No | 8,615 | 99.7 |
| Valvular disease (non-rheumatic) |  |  |
| Yes | 80 | 0.9 |
| No | 8,565 | 99.1 |
| Heart failure |  |  |
| Yes | 167 | 1.9 |
| No | 8,478 | 98.1 |
| Conduction disorders |  |  |
| Yes | 167 | 1.9 |
| No | 8,478 | 98.1 |
| Cerebrovascular disease |  |  |
| Yes | 182 | 2.1 |
| No | 8,463 | 97.9 |
| Diseases of arteries, arterioles, and capillaries | |  |
| Yes | 77 | 0.9 |
| No | 8,568 | 99.1 |
| Venous and lymphatic disease |  |  |
| Yes | 145 | 1.7 |
| No | 8,500 | 98.3 |
| Other complications of the circulatory system | |  |
| Yes | 50 | 0.6 |
| No | 8,595 | 99.4 |
| Total | 8,645 | 100.0 |
| To protect confidentiality and minimize disclosure risk, sub-diseases with small cell counts (n ≤ 5) have been omitted from frequency tables. | | |

**Online Resource Table 11**. Burden of respiratory disease in the last 5 years among long-term childhood cancer survivors

|  | No. | % |  |
| --- | --- | --- | --- |
| Influenza |  |  |  |
| Yes | 63 | 0.7 |  |
| No | 8,582 | 99.3 |  |
| Acute upper respiratory infections |  |  |  |
| Yes | 340 | 3.9 |  |
| No | 8,305 | 96.1 |  |
| Other disorders of the upper respiratory tract | |  |  |
| Yes | 485 | 5.6 |  |
| No | 8,160 | 94.4 |  |
| Pneumonia |  |  |  |
| Yes | 194 | 2.2 |  |
| No | 8,451 | 97.8 |  |
| Abscess of lung and pyothorax |  |  |  |
| Yes | 36 | 0.4 |  |
| No | 8,609 | 99.6 |  |
| Bronchitis and emphysema |  |  |  |
| Yes | 78 | 0.9 |  |
| No | 8,567 | 99.1 |  |
| Asthma |  |  |  |
| Yes | 292 | 3.4 |  |
| No | 8,353 | 96.6 |  |
| Lung diseases due to external agents |  |  |  |
| Yes | 32 | 0.4 |  |
| No | 8,613 | 99.6 |  |
| Interstitial pulmonary diseases and pulmonary oedema | |  |  |
| Yes | 16 | 0.2 |  |
| No | 8,629 | 99.8 |  |
| Pneumothorax |  |  |  |
| Yes | 17 | 0.2 |  |
| No | 8,628 | 99.8 |  |
| Respiratory failure |  |  |  |
| Yes | 52 | 0.6 |  |
| No | 8,593 | 99.4 |  |
| Other diseases of respiratory system |  |  |  |
| Yes | 39 | 0.5 |  |
| No | 8,606 | 99.5 |  |
| Total | 8,645 | 100.0 |  |
| To protect confidentiality and minimize disclosure risk, sub-diseases with small cell counts (n ≤ 5) have been omitted from frequency tables. | | | |

**Online Resource Table 12**. Burden of digestive disease in the last 5 years among long-term childhood cancer survivors

|  | No. | % |  |
| --- | --- | --- | --- |
| Diseases of the teeth and supporting structures | |  |  |
| Yes | 129 | 1.5 |  |
| No | 8,516 | 98.5 |  |
| Other diseases of the oral cavity and salivary glands | |  |  |
| Yes | 112 | 1.3 |  |
| No | 8,533 | 98.7 |  |
| Diseases of the esophagus |  |  |  |
| Yes | 117 | 1.4 |  |
| No | 8,528 | 98.6 |  |
| Diseases of the stomach and duodenum |  |  |  |
| Yes | 162 | 1.9 |  |
| No | 8,483 | 98.1 |  |
| Appendicitis |  |  |  |
| Yes | 70 | 0.8 |  |
| No | 8,575 | 99.2 |  |
| Hernia of the abdominal cavity |  |  |  |
| Yes | 115 | 1.3 |  |
| No | 8,530 | 98.7 |  |
| Non-infective enteritis and colitis |  |  |  |
| Yes | 119 | 1.4 |  |
| No | 8,526 | 98.6 |  |
| Paralytic ileus and intestinal obstruction | |  |  |
| Yes | 64 | 0.7 |  |
| No | 8,581 | 99.3 |  |
| Diseases of the anal and rectal regions |  |  |  |
| Yes | 93 | 1.1 |  |
| No | 8,552 | 98.9 |  |
| Diseases of the peritoneum |  |  |  |
| Yes | 24 | 0.3 |  |
| No | 8,621 | 99.7 |  |
| Other diseases of the digestive system |  |  |  |
| Yes | 545 | 6.3 |  |
| No | 8,100 | 93.7 |  |
| Diseases of the liver |  |  |  |
| Yes | 55 | 0.6 |  |
| No | 8,590 | 99.4 |  |
| Diseases of the gallbladder and biliary ducts | |  |  |
| Yes | 123 | 1.4 |  |
| No | 8,522 | 98.6 |  |
| Diseases of the pancreas |  |  |  |
| Yes | 44 | 0.5 |  |
| No | 8,601 | 99.5 |  |
| Total | 8,645 | 100.0 |  |
| To protect confidentiality and minimize disclosure risk, sub-diseases with small cell counts (n ≤ 5) have been omitted from frequency tables. | | | |

**Online Resource Table 13**. Burden of urinary and genital diseases in the last 5 years among long-term childhood cancer survivors

|  | No. | % |  |
| --- | --- | --- | --- |
| Glomerular Disease |  |  |  |
| Yes | 30 | 0.3 |  |
| No | 8,615 | 99.7 |  |
| Acute renal failure |  |  |  |
| Yes | 57 | 0.7 |  |
| No | 8,588 | 99.3 |  |
| Chronic kidney disease |  |  |  |
| Yes | 231 | 2.7 |  |
| No | 8,414 | 97.3 |  |
| Urolithiasis |  |  |  |
| Yes | 102 | 1.2 |  |
| No | 8,543 | 98.8 |  |
| Obstructive uropathy |  |  |  |
| Yes | 57 | 0.7 |  |
| No | 8,588 | 99.3 |  |
| Infections of the urinary system |  |  |  |
| Yes | 290 | 3.4 |  |
| No | 8,355 | 96.6 |  |
| Other and unspecified disorders of the urinary system | |  |  |
| Yes | 162 | 1.9 |  |
| No | 8,483 | 98.1 |  |
| Diseases of the prostate |  |  |  |
| Yes | 36 | 0.4 |  |
| No | 8,609 | 99.6 |  |
| Hydrocoele and spermatocoele |  |  |  |
| Yes | 17 | 0.2 |  |
| No | 8,628 | 99.8 |  |
| Orchitis and epididymitis |  |  |  |
| Yes | 31 | 0.4 |  |
| No | 8,614 | 99.6 |  |
| Other diseases of the male genital organs | |  |  |
| Yes | 138 | 1.6 |  |
| No | 8,507 | 98.4 |  |
| Chronic cystic disease and other diseases of the breast | | |  |
| Yes | 63 | 0.7 |  |
| No | 8,582 | 99.3 |  |
| Inflammatory diseases of the female pelvic organs | |  |  |
| Yes | 174 | 2.0 |  |
| No | 8,471 | 98.0 |  |
| Endometriosis |  |  |  |
| Yes | 32 | 0.4 |  |
| No | 8,613 | 99.6 |  |
| Noninflammatory disorders of the female genital tract | |  |  |
| Yes | 356 | 4.1 |  |
| No | 8,289 | 95.9 |  |
| Male sterility |  |  |  |
| Yes | 105 | 1.2 |  |
| No | 8,540 | 98.8 |  |
| Abnormal menstruation |  |  |  |
| Yes | 308 | 3.6 |  |
| No | 8,337 | 96.4 |  |
| Female infertility |  |  |  |
| Yes | 122 | 1.4 |  |
| No | 8,523 | 98.6 |  |
| Other disorders of the female reproductive system | |  |  |
| Yes | 386 | 4.5 |  |
| No | 8,259 | 95.5 |  |
| Total | 8,645 | 100.0 |  |
| To protect confidentiality and minimize disclosure risk, sub-diseases with small cell counts (n ≤ 5) have been omitted from frequency tables. | | | |

**Online Resource Table 14**. Burden of skin diseases in the last 5 years among long-term childhood cancer survivors

|  | No. | % |
| --- | --- | --- |
| Infections of the skin and subcutaneous tissue | |  |
| Yes | 259 | 3.0 |
| No | 8,386 | 97.0 |
| Other inflammatory conditions of the skin and subcutaneous tissue | | |
| Yes | 613 | 7.1 |
| No | 8,032 | 92.9 |
| Disorders of skin appendages (hair, nails, sweat glands) | | |
| Yes | 352 | 4.1 |
| No | 8,293 | 95.9 |
| Other disorders of the skin and subcutaneous tissue | |  |
| Yes | 464 | 5.4 |
| No | 8,181 | 94.6 |
| Total | 8,645 | 100.0 |
| To protect confidentiality and minimize disclosure risk, sub-diseases with small cell counts (n ≤ 5) have been omitted from frequency tables. | | |

**Online Resource Table 15**. Burden of bone, joint, and muscle diseases in the last 5 years among long-term childhood cancer survivors

|  | No. | % |
| --- | --- | --- |
| Arthritis and rheumatism |  |  |
| Yes | 285 | 3.3 |
| No | 8,360 | 96.7 |
| Osteomyelitis and other diseases of bone and joints | |  |
| Yes | 992 | 11.5 |
| No | 7,653 | 88.5 |
| Other diseases of the musculoskeletal system | |  |
| Yes | 903 | 10.4 |
| No | 7,742 | 89.6 |
| Total | 8,645 | 100.0 |
| To protect confidentiality and minimize disclosure risk, sub-diseases with small cell counts (n ≤ 5) have been omitted from frequency tables. | | |

**Online Resource Table 16**. Recent disease burden among long-term childhood cancer survivors diagnosed between 1958 and 1990, by cancer type

|  | **Cancer type (Diagnosed 1958-1990)** | | | | | | | | | |
| --- | --- | --- | --- | --- | --- | --- | --- | --- | --- | --- |
|  | **Leukemias** | | **Lymphomas** | | **CNS tumors** | | **Other solid tumors** | | **Overall** | |
|  | No. | % | No. | % | No. | % | No. | % | No. | % |
| **Number of diseases** | |  |  |  |  |  |  |  |  |  |
| 0 | 162 | 26.6 | 79 | 25.1 | 192 | 21.1 | 317 | 27.6 | 750 | 25.2 |
| 1-2 | 255 | 41.9 | 141 | 44.8 | 363 | 40.0 | 460 | 40.0 | 1,219 | 40.9 |
| 3 or more | 191 | 31.4 | 95 | 30.2 | 353 | 38.9 | 372 | 32.4 | 1,011 | 33.9 |
| **Infectious or parasitic disease** | | |  |  |  |  |  |  |  |  |
| Yes | 43 | 7.1 | 27 | 8.6 | 104 | 11.5 | 89 | 7.7 | 263 | 8.8 |
| No | 565 | 92.9 | 288 | 91.4 | 804 | 88.5 | 1,060 | 92.3 | 2,717 | 91.2 |
| **Benign tumor** | | |  |  |  |  |  |  |  |  |
| Yes | 86 | 14.1 | 50 | 15.9 | 98 | 10.8 | 157 | 13.7 | 391 | 13.1 |
| No | 522 | 85.9 | 265 | 84.1 | 810 | 89.2 | 992 | 86.3 | 2,589 | 86.9 |
| **Blood disease** | | |  |  |  |  |  |  |  |  |
| Yes | 23 | 3.8 | 12 | 3.8 | 45 | 5.0 | 57 | 5.0 | 137 | 4.6 |
| No | 585 | 96.2 | 303 | 96.2 | 863 | 95.0 | 1,092 | 95.0 | 2,843 | 95.4 |
| **Endocrine, nutritional, or metabolic disease** | | | |  |  |  |  |  |  |  |
| Yes | 159 | 26.2 | 63 | 20.0 | 227 | 25.0 | 204 | 17.8 | 653 | 21.9 |
| No | 449 | 73.8 | 252 | 80.0 | 681 | 75.0 | 945 | 82.2 | 2,327 | 78.1 |
| **Mental or behavioral disease** | | |  |  |  |  |  |  |  |  |
| Yes | 82 | 13.5 | 33 | 10.5 | 162 | 17.8 | 116 | 10.1 | 393 | 13.2 |
| No | 526 | 86.5 | 282 | 89.5 | 746 | 82.2 | 1,033 | 89.9 | 2,587 | 86.8 |
| **Diseases of the nervous system** | | | |  |  |  |  |  |  |  |
| Yes | 79 | 13.0 | 24 | 7.6 | 279 | 30.7 | 123 | 10.7 | 505 | 16.9 |
| No | 529 | 87.0 | 291 | 92.4 | 629 | 69.3 | 1,026 | 89.3 | 2,475 | 83.1 |
| **Disease of the eye or ear** | | |  |  |  |  |  |  |  |  |
| Yes | 123 | 20.2 | 44 | 14.0 | 266 | 29.3 | 272 | 23.7 | 705 | 23.7 |
| No | 485 | 79.8 | 271 | 86.0 | 642 | 70.7 | 877 | 76.3 | 2,275 | 76.3 |
| **Disease of the circulatory system** | | | |  |  |  |  |  |  |  |
| Yes | 117 | 19.2 | 78 | 24.8 | 188 | 20.7 | 244 | 21.2 | 627 | 21.0 |
| No | 491 | 80.8 | 237 | 75.2 | 720 | 79.3 | 905 | 78.8 | 2,353 | 79.0 |
| **Disease of the respiratory system** | | | |  |  |  |  |  |  |  |
| Yes | 58 | 9.5 | 42 | 13.3 | 101 | 11.1 | 116 | 10.1 | 317 | 10.6 |
| No | 550 | 90.5 | 273 | 86.7 | 807 | 88.9 | 1,033 | 89.9 | 2,663 | 89.4 |
| **Disease of the digestive system** | | |  |  |  |  |  |  |  |  |
| Yes | 79 | 13.0 | 51 | 16.2 | 135 | 14.9 | 186 | 16.2 | 451 | 15.1 |
| No | 529 | 87.0 | 264 | 83.8 | 773 | 85.1 | 963 | 83.8 | 2,529 | 84.9 |
| **Disease of the urinary system or genitals** | | | | |  |  |  |  |  |  |
| Yes | 161 | 26.5 | 62 | 19.7 | 223 | 24.6 | 270 | 23.5 | 716 | 24.0 |
| No | 447 | 73.5 | 253 | 80.3 | 685 | 75.4 | 879 | 76.5 | 2,264 | 76.0 |
| **Disease of the skin and subcutaneous tissues** | | | | |  |  |  |  |  |  |
| Yes | 88 | 14.5 | 51 | 16.2 | 145 | 16.0 | 167 | 14.5 | 451 | 15.1 |
| No | 520 | 85.5 | 264 | 83.8 | 763 | 84.0 | 982 | 85.5 | 2,529 | 84.9 |
| **Disease of the bones, joints, and muscles** | | | |  |  |  |  |  |  |  |
| Yes | 113 | 18.6 | 69 | 21.9 | 219 | 24.1 | 293 | 25.5 | 694 | 23.3 |
| No | 495 | 81.4 | 246 | 78.1 | 689 | 75.9 | 856 | 74.5 | 2,286 | 76.7 |
| Total | 608 | 100.0 | 315 | 100.0 | 908 | 100.0 | 1,149 | 100.0 | 2,980 | 100.0 |

Disease burden in the 5-year period preceding December 31^st^, 2023 among long-term childhood cancer survivors (diagnosed ages 0-14, 1958-1990, survived >=5 years from diagnosis) registered as alive and living in Sweden on December 31^st^, 2023, by cancer type. Disease burden reflects diagnoses recorded in outpatient specialist care and inpatient care. Diagnoses recorded in primary care are not included.

**Online Resource Table 17**. Recent disease burden among long-term childhood cancer survivors diagnosed between 1991 and 2018, by cancer type

|  | **Cancer type (Diagnosed 1991-2018)** | | | | | | | | | |
| --- | --- | --- | --- | --- | --- | --- | --- | --- | --- | --- |
|  | **Leukemias** | | **Lymphomas** | | **CNS tumors** | | **Other solid tumors** | | **Overall** | |
|  | No. | % | No. | % | No. | % | No. | % | No. | % |
| **Number of diseases** | |  |  |  |  |  |  |  |  |  |
| 0 | 508 | 27.5 | 205 | 33.2 | 278 | 19.5 | 431 | 24.3 | 1,422 | 25.1 |
| 1-2 | 814 | 44.0 | 267 | 43.3 | 581 | 40.7 | 774 | 43.7 | 2,436 | 43.0 |
| 3 or more | 527 | 28.5 | 145 | 23.5 | 569 | 39.8 | 566 | 32.0 | 1,807 | 31.9 |
| **Infectious or parasitic disease** | |  |  |  |  |  |  |  |  |  |
| Yes | 233 | 12.6 | 77 | 12.5 | 138 | 9.7 | 211 | 11.9 | 659 | 11.6 |
| No | 1,616 | 87.4 | 540 | 87.5 | 1,290 | 90.3 | 1,560 | 88.1 | 5,006 | 88.4 |
| **Benign tumor** | |  |  |  |  |  |  |  |  |  |
| Yes | 149 | 8.1 | 59 | 9.6 | 139 | 9.7 | 197 | 11.1 | 544 | 9.6 |
| No | 1,700 | 91.9 | 558 | 90.4 | 1,289 | 90.3 | 1,574 | 88.9 | 5,121 | 90.4 |
| **Blood disease** |  |  |  |  |  |  |  |  |  |  |
| Yes | 107 | 5.8 | 29 | 4.7 | 62 | 4.3 | 73 | 4.1 | 271 | 4.8 |
| No | 1,742 | 94.2 | 588 | 95.3 | 1,366 | 95.7 | 1,698 | 95.9 | 5,394 | 95.2 |
| **Endocrine, nutritional, or metabolic disease** | | |  |  |  |  |  |  |  |  |
| Yes | 382 | 20.7 | 111 | 18.0 | 451 | 31.6 | 368 | 20.8 | 1,312 | 23.2 |
| No | 1,467 | 79.3 | 506 | 82.0 | 977 | 68.4 | 1,403 | 79.2 | 4,353 | 76.8 |
| **Mental or behavioral disease** | | |  |  |  |  |  |  |  |  |
| Yes | 364 | 19.7 | 113 | 18.3 | 438 | 30.7 | 393 | 22.2 | 1,308 | 23.1 |
| No | 1,485 | 80.3 | 504 | 81.7 | 990 | 69.3 | 1,378 | 77.8 | 4,357 | 76.9 |
| **Diseases of the nervous system** | |  |  |  |  |  |  |  |  |  |
| Yes | 176 | 9.5 | 42 | 6.8 | 481 | 33.7 | 191 | 10.8 | 890 | 15.7 |
| No | 1,673 | 90.5 | 575 | 93.2 | 947 | 66.3 | 1,580 | 89.2 | 4,775 | 84.3 |
| **Disease of the eye or ear** | |  |  |  |  |  |  |  |  |  |
| Yes | 372 | 20.1 | 70 | 11.3 | 517 | 36.2 | 414 | 23.4 | 1,373 | 24.2 |
| No | 1,477 | 79.9 | 547 | 88.7 | 911 | 63.8 | 1,357 | 76.6 | 4,292 | 75.8 |
| **Disease of the circulatory system** | |  |  |  |  |  |  |  |  |  |
| Yes | 137 | 7.4 | 45 | 7.3 | 105 | 7.4 | 134 | 7.6 | 421 | 7.4 |
| No | 1,712 | 92.6 | 572 | 92.7 | 1,323 | 92.6 | 1,637 | 92.4 | 5,244 | 92.6 |
| **Disease of the respiratory system** | | |  |  |  |  |  |  |  |  |
| Yes | 325 | 17.6 | 86 | 13.9 | 171 | 12.0 | 250 | 14.1 | 832 | 14.7 |
| No | 1,524 | 82.4 | 531 | 86.1 | 1,257 | 88.0 | 1,521 | 85.9 | 4,833 | 85.3 |
| **Disease of the digestive system** | |  |  |  |  |  |  |  |  |  |
| Yes | 276 | 14.9 | 78 | 12.6 | 180 | 12.6 | 301 | 17.0 | 835 | 14.7 |
| No | 1,573 | 85.1 | 539 | 87.4 | 1,248 | 87.4 | 1,470 | 83.0 | 4,830 | 85.3 |
| **Disease of the urinary system or genitals** | | |  |  |  |  |  |  |  |  |
| Yes | 325 | 17.6 | 102 | 16.5 | 238 | 16.7 | 457 | 25.8 | 1,122 | 19.8 |
| No | 1,524 | 82.4 | 515 | 83.5 | 1,190 | 83.3 | 1,314 | 74.2 | 4,543 | 80.2 |
| **Disease of the skin and subcutaneous tissues** | | | |  |  |  |  |  |  |  |
| Yes | 329 | 17.8 | 95 | 15.4 | 241 | 16.9 | 264 | 14.9 | 929 | 16.4 |
| No | 1,520 | 82.2 | 522 | 84.6 | 1,187 | 83.1 | 1,507 | 85.1 | 4,736 | 83.6 |
| **Disease of the bones, joints, and muscles** | | |  |  |  |  |  |  |  |  |
| Yes | 308 | 16.7 | 100 | 16.2 | 273 | 19.1 | 340 | 19.2 | 1,021 | 18.0 |
| No | 1,541 | 83.3 | 517 | 83.8 | 1,155 | 80.9 | 1,431 | 80.8 | 4,644 | 82.0 |
| Total | 1,849 | 100.0 | 617 | 100.0 | 1,428 | 100.0 | 1,771 | 100.0 | 5,665 | 100.0 |

Disease burden in the 5-year period preceding December 31^st^, 2023 among long-term childhood cancer survivors (diagnosed ages 0-14, 1991-2018, survived >=5 years from diagnosis) registered as alive and living in Sweden on December 31^st^, 2023, by cancer type. Disease burden reflects diagnoses recorded in outpatient specialist care and inpatient care. Diagnoses recorded in primary care are not included.

**Online Resource Table 18.** Household income distribution of long-term central nervous system tumor survivors, by attained age in 2023

|  | **Age on December 31^st^, 2023 (years)** | | | | | | | | | | | |
| --- | --- | --- | --- | --- | --- | --- | --- | --- | --- | --- | --- | --- |
| **Household income quartile** | **19-25** | | **26-35** | | **36-45** | | **46-55** | | **>55** | | **Overall** | |
|  | No. | % | No. | % | No. | % | No. | % | No. | % | No. | % |
| Q1 (lowest) | 74 | 23.4 | 169 | 33.3 | 140 | 30.1 | 146 | 41.0 | 130 | 40.8 | 659 | 33.6 |
| Q2 | 55 | 17.4 | 130 | 25.6 | 136 | 29.2 | 78 | 21.9 | 78 | 24.5 | 477 | 24.3 |
| Q3 | 84 | 26.6 | 112 | 22.0 | 103 | 22.2 | 80 | 22.5 | 58 | 18.2 | 437 | 22.3 |
| Q4 (highest) | 103 | 32.6 | 97 | 19.1 | 86 | 18.5 | 52 | 14.6 | 53 | 16.6 | 391 | 19.9 |
| Total | 316 | 100.0 | 508 | 100.0 | 465 | 100.0 | 356 | 100.0 | 319 | 100.0 | 1,964 | 100.0 |

Includes individuals diagnosed with a central nervous system tumor between the ages 0-14 years from 1991-2018 who survived >=5 years from diagnosis and were registered as alive and living in Sweden on December 31^st^, 2023, Income quartiles were constructed using the general population’s income distribution, adjusted for household size and age group.

**Online Resource Table 19**. Prevalence of long-term childhood cancer survivors in Sweden between 1990-2023.

| Observed Prevalence | |
| --- | --- |
| Year | Number |
| 1990 | 2653 |
| 1991 | 2819 |
| 1992 | 2975 |
| 1993 | 3151 |
| 1994 | 3308 |
| 1995 | 3476 |
| 1996 | 3640 |
| 1997 | 3843 |
| 1998 | 4019 |
| 1999 | 4205 |
| 2000 | 4389 |
| 2001 | 4595 |
| 2002 | 4794 |
| 2003 | 5001 |
| 2004 | 5164 |
| 2005 | 5322 |
| 2006 | 5497 |
| 2007 | 5679 |
| 2008 | 5873 |
| 2009 | 6051 |
| 2010 | 6230 |
| 2011 | 6410 |
| 2012 | 6581 |
| 2013 | 6741 |
| 2014 | 6911 |
| 2015 | 7118 |
| 2016 | 7313 |
| 2017 | 7523 |
| 2018 | 7742 |
| 2019 | 7945 |
| 2020 | 8127 |
| 2021 | 8311 |
| 2022 | 8509 |
| 2023 | 8645 |

Online Resource Table 19 displays the observed prevalence of long-term childhood cancer survivors (diagnosed at ages 0–14 years, from 1958–2018, ≥5 years post-diagnosis) on December 31^st^ of each year from 1990-2023. Note that the duration of prevalence is different in each year from 1990-2023. In 1990, there was a 42-year surveillance period and the surveillance period increased by one each year. Accordingly, the surveillance period in 2023 was 65 years. Comparisons across time should consider the changing definition of prevalence across this time.

**Online Resource Table 20**. Projected prevalence of long-term childhood cancer survivors living in Sweden, 2024-2040, across different scenarios

Online Resource Table 20 displays the projected prevalence of long-term childhood cancer survivors (diagnosed at ages 0–14 years, from 1958–2018, ≥5 years post-diagnosis) from 2024-2040 under different scenarios of 5-year survival and long-term mortality. Projected scenarios and their corresponding assumptions are as follows:

1. Stable short-term (5-year) survival and long-term mortality rates of the general Swedish population.

2. Stable short-term (5-year) survival and excess long-term mortality.

3. Improving short-term (5-year) survival and long-term mortality rates of the general Swedish population.

4. Improving short-term (5-year) survival and excess long-term mortality.

All scenarios assume stable incidence.

Online Resource Table 20. Projected prevalence of long-term childhood cancer survivors living in Sweden, 2024-2040, across different scenarios

|  | Projected Prevalence | | | |
| --- | --- | --- | --- | --- |
| Year | Scenario 1 | Scenario 2 | Scenario 3 | Scenario 4 |
| 2024 | 8900 | 8900 | 8900 | 8900 |
| 2025 | 9200 | 9100 | 9200 | 9100 |
| 2026 | 9400 | 9300 | 9400 | 9300 |
| 2027 | 9700 | 9500 | 9700 | 9500 |
| 2028 | 9900 | 9700 | 9900 | 9700 |
| 2029 | 10200 | 9900 | 10200 | 9900 |
| 2030 | 10400 | 10100 | 10400 | 10100 |
| 2031 | 10600 | 10200 | 10600 | 10300 |
| 2032 | 10800 | 10400 | 10900 | 10400 |
| 2033 | 11100 | 10600 | 11100 | 10600 |
| 2034 | 11300 | 10700 | 11300 | 10700 |
| 2035 | 11500 | 10800 | 11500 | 10900 |
| 2036 | 11700 | 11000 | 11700 | 11000 |
| 2037 | 11900 | 11100 | 12000 | 11200 |
| 2038 | 12100 | 11200 | 12200 | 11300 |
| 2039 | 12300 | 11300 | 12400 | 11400 |
| 2040 | 12500 | 11400 | 12600 | 11500 |

|  |  |  | **Age (on December 31^st^ of projection year)** | | | | | | | |
| --- | --- | --- | --- | --- | --- | --- | --- | --- | --- | --- |
|  | **Overall** | | **5-18** | | **19-35** | | **36-55** | | **>55** | |
| **Projection Year** | Scenario 2 | Scenario 3 | Scenario 2 | Scenario 3 | Scenario 2 | Scenario 3 | Scenario 2 | Scenario 3 | Scenario 2 | Scenario 3 |
| 2024 | 8900 | 8900 | 1800 | 1800 | 3300 | 3400 | 2800 | 2800 | 900 | 900 |
| 2025 | 9100 | 9200 | 1800 | 1800 | 3400 | 3400 | 2900 | 2900 | 1000 | 1000 |
| 2026 | 9300 | 9400 | 1900 | 1900 | 3400 | 3400 | 2900 | 3000 | 1100 | 1200 |
| 2027 | 9500 | 9700 | 1900 | 1900 | 3400 | 3400 | 3000 | 3100 | 1200 | 1300 |
| 2028 | 9700 | 9900 | 1900 | 1900 | 3400 | 3500 | 3100 | 3200 | 1300 | 1400 |
| 2029 | 9900 | 10200 | 1900 | 1900 | 3400 | 3500 | 3200 | 3300 | 1400 | 1500 |
| 2030 | 10100 | 10400 | 1900 | 1900 | 3500 | 3600 | 3300 | 3400 | 1500 | 1600 |
| 2031 | 10200 | 10600 | 1900 | 1900 | 3500 | 3600 | 3300 | 3400 | 1600 | 1700 |
| 2032 | 10400 | 10900 | 1900 | 1900 | 3600 | 3700 | 3300 | 3400 | 1700 | 1800 |
| 2033 | 10600 | 11100 | 1900 | 1900 | 3600 | 3700 | 3400 | 3500 | 1700 | 1900 |
| 2034 | 10700 | 11300 | 1900 | 1900 | 3600 | 3800 | 3400 | 3500 | 1800 | 2000 |
| 2035 | 10800 | 11500 | 1900 | 1900 | 3700 | 3900 | 3400 | 3600 | 1900 | 2200 |
| 2036 | 11000 | 11700 | 1900 | 1900 | 3700 | 3900 | 3500 | 3600 | 1900 | 2300 |
| 2037 | 11100 | 12000 | 1800 | 1900 | 3700 | 4000 | 3500 | 3700 | 2000 | 2400 |
| 2038 | 11200 | 12200 | 1800 | 1900 | 3700 | 4000 | 3500 | 3700 | 2100 | 2500 |
| 2039 | 11300 | 12400 | 1800 | 1800 | 3800 | 4100 | 3500 | 3700 | 2200 | 2700 |
| 2040 | 11400 | 12600 | 1800 | 1800 | 3800 | 4200 | 3600 | 3800 | 2200 | 2800 |

**Online Resource Table 21**. Projected prevalence of long-term childhood cancer survivors living in Sweden by attained age, 2024-2040 (Scenarios 2 and 3)

Scenario 2 corresponds to stable short-term (5-year) mortality and excess long-term mortality; scenario 3 corresponds to improving (5-year) mortality and long-term mortality rates of the general Swedish population.

**Online Resource Figure 2**. Projected prevalence of long-term childhood cancer survivors living in Sweden by attained age, 2024-2040, assuming improving (5-year) mortality and long-term mortality rates of the general Swedish population


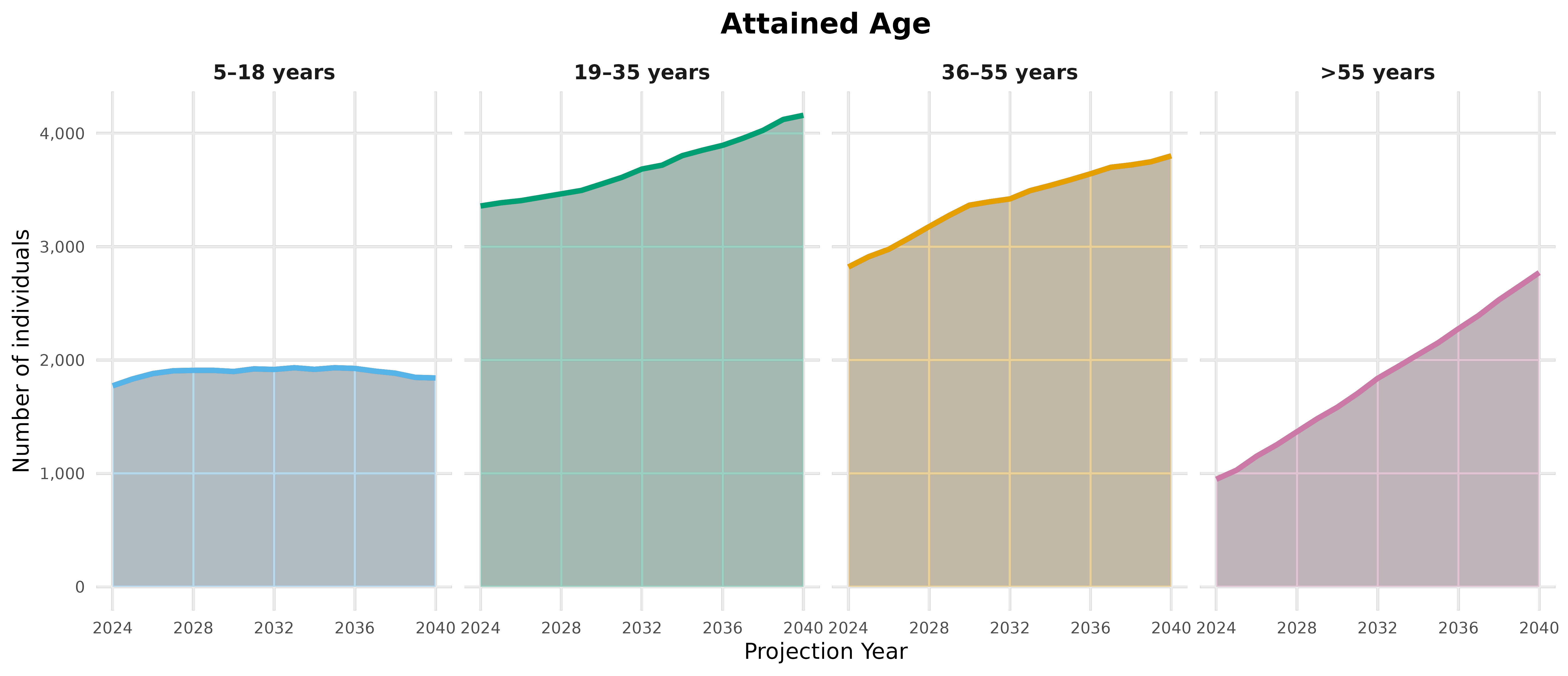


**Online Resource References**

1. Steliarova-Foucher E, Stiller C, Lacour B, Kaatsch P. International Classification of Childhood Cancer, third edition. *Cancer*. 2005;103(7):1457-1467. doi:10.1002/cncr.20910

2. Steliarova-Foucher E, Colombet M, Ries L, Rous B, Stiller C. International Classification of Childhood Cancer, 3rd edition, update 2017. In: *International Incidence of Childhood Cancer, Volume 3*. International Agency for Research on Cancer. Accessed August 4, 2025. https://iicc.iarc.fr/classification/diagnostic-groups-and-subgroups/

3. The National Board of Health and Welfare. *National Cancer Register*. Socialstyrelsen; 2023. https://www.socialstyrelsen.se/globalassets/sharepoint-dokument/dokument-webb/ovrigt/production-and-quality-can.pdf

4. de Fine Licht S, Rugbjerg K, Gudmundsdottir T, et al. Long-term inpatient disease burden in the Adult Life after Childhood Cancer in Scandinavia (ALiCCS) study: A cohort study of 21,297 childhood cancer survivors. *PLoS Med*. 2017;14(5):e1002296. doi:10.1371/journal.pmed.1002296

5. Frederiksen LE, Erdmann F, Mader L, et al. Psychiatric disorders in childhood cancer survivors in Denmark, Finland, and Sweden: a register-based cohort study from the SALiCCS research programme. *Lancet Psychiatry*. 2022;9(1):35-45. doi:10.1016/S2215-0366(21)00387-4

6. National Board of Health and Welfare. *National Patient Register*. National Board of Health and Welfare; 2023. https://www.socialstyrelsen.se/globalassets/sharepoint-dokument/dokument-webb/statistik/production-and-quality-of-the-patient-register.pdf

7. National Board of Health and Welfare. Statistical Database, Cancer. Published online January 31, 2025. Accessed June 17, 2025. https://sdb.socialstyrelsen.se/if_can/val_eng.aspx

8. Statistics Sweden. Statistical database, population. Published online April 25, 2024. Accessed June 16, 2025. https://www.statistikdatabasen.scb.se/pxweb/en/ssd/START__BE__BE0401/

9. Moskalewicz A, Martinez B, Uleryk EM, Pechlivanoglou P, Gupta S, Nathan PC. Late mortality among 5-year survivors of childhood cancer: A systematic review and meta-analysis. *Cancer*. 2024;130(10):1844-1857. doi:10.1002/cncr.35213

10. Bagnasco F, Caruso S, Andreano A, et al. Late mortality and causes of death among 5-year survivors of childhood cancer diagnosed in the period 1960–1999 and registered in the Italian Off-Therapy Registry. *Eur J Cancer*. 2019;110:86-97. doi:10.1016/j.ejca.2018.12.021

11. Byrne J, Schmidtmann I, Rashid H, et al. Impact of era of diagnosis on cause-specific late mortality among 77 423 five-year European survivors of childhood and adolescent cancer: The PanCareSurFup consortium. *Int J Cancer*. 2022;150(3):406-419. doi:10.1002/ijc.33817

12. Cardous-Ubbink M c., Heinen R c., Langeveld N e., et al. Long-term cause-specific mortality among five-year survivors of childhood cancer. *Pediatr Blood Cancer*. 2004;42(7):563-573. doi:10.1002/pbc.20028

13. Dixon SB, Liu Q, Chow EJ, et al. Specific causes of excess late mortality and association with modifiable risk factors among survivors of childhood cancer: a report from the Childhood Cancer Survivor Study cohort. *The Lancet*. 2023;401(10386):1447-1457. doi:10.1016/S0140-6736(22)02471-0

14. Ehrhardt MJ, Liu Q, Dixon SB, et al. Association of Modifiable Health Conditions and Social Determinants of Health With Late Mortality in Survivors of Childhood Cancer. *JAMA Netw Open*. 2023;6(2):e2255395. doi:10.1001/jamanetworkopen.2022.55395

15. Fidler MM, Reulen RC, Winter DL, et al. Long term cause specific mortality among 34 489 five year survivors of childhood cancer in Great Britain: population based cohort study. *BMJ*. 2016;354:i4351. doi:10.1136/bmj.i4351

16. MacArthur AC, Spinelli JJ, Rogers PC, Goddard KJ, Abanto ZU, McBride ML. Mortality among 5-year survivors of cancer diagnosed during childhood or adolescence in British Columbia, Canada. *Pediatr Blood Cancer*. 2007;48(4):460-467. doi:10.1002/pbc.20922

17. Schindler M, Spycher BD, Ammann RA, et al. Cause-specific long-term mortality in survivors of childhood cancer in Switzerland: A population-based study. *Int J Cancer*. 2016;139(2):322-333. doi:10.1002/ijc.30080

18. Wilson CL, Cohn RJ, Johnston KA, Ashton LJ. Late mortality and second cancers in an Australian cohort of childhood cancer survivors. *Med J Aust*. 2010;193(5):258-261. doi:10.5694/j.1326-5377.2010.tb03902.x
